# A water-soluble membrane transporter for biologically relevant cations†

**DOI:** 10.1039/d2ra05314d

**Published:** 2022-09-29

**Authors:** Kylie Yang, Jessica E. Boles, Lisa J. White, Kira L. F. Hilton, Hin Yuk Lai, Yifan Long, Jennifer R. Hiscock, Cally J. E. Haynes

**Affiliations:** Chemistry Department, UCL 20 Gordon Street London WC1H 0AJ UK cally.haynes@ucl.ac.uk; School of Chemistry and Forensic Science, University of Kent Canterbury Kent CT2 7NH UK j.r.hiscock@kent.ac.uk

## Abstract

Synthetic ionophores are promising therapeutic targets, yet poor water solubility limits their potential for translation into the clinic. Here we report a water-soluble, supramolecular self-associating amphiphile that functions as a cation uniporter in synthetic vesicle systems, deriving mechanistic insight through planar bilayer patch clamp experiments.

## Abstract 2

Synthetic ionophores are promising therapeutic targets, yet poor water solubility limits their potential for translation into the clinic. Here we report a water soluble, supramolecular self-associating amphiphile (SSA) with cation transport function.

## Graphical abstract

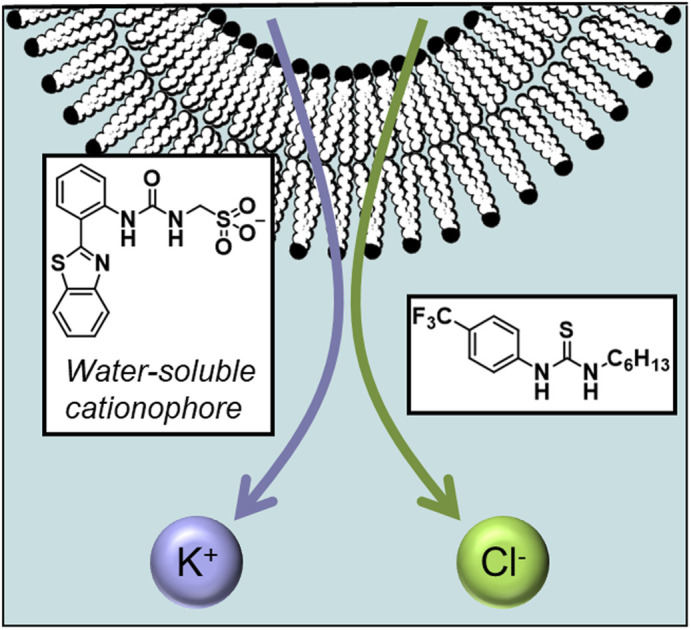

## Introduction

Synthetic ion transporters^1–6^ have been developed for a range of therapeutic applications, such as the treatment of cancer, microbial infections and channelopathies.^7,8^ Work in this field to date has meant that it is now possible to rationally design potent synthetic ionophores with excellent transport activity in synthetic vesicle systems. However, the poor water solubility associated with these agents means that biological deliverability^9^ remains one of the major challenges facing the successful translation of this technology into the clinic.

High lipophilicity and ion binding strength have been shown to increase ion transport activity.^10^ However, high molecular lipophilicity is also known to lower water solubility, decreasing the concentration of ion transporter present in physiologically relevant fluids, resulting in a compound which exhibits limited drug development prospects. Poor transporter water-solubility has hampered progress in clinical trials.^11^ Furthermore, highly lipophilic therapeutics can display off–target interactions (*e.g.* with hydrophobic proteins)^12^ and form large aggregates which limit molecular diffusion, preventing the agent reaching the desired site of action in a high enough concentration to elicit a therapeutic effect.^13^

To maximise the therapeutic potential of synthetic ionophores, a strategy to enable both deliverability and potency is required. Recent work to improve the delivery of ionophores to the site of therapeutic action have included encapsulation within synthetic phospholipid vesicles,^9^ pro-drug strategies^14,15^ and complexation within cyclodextrins.^16^ However, the development of intrinsically water-soluble transporters remains the ideal solution to enable line-of-site to the clinic.

**Fig. 1:**
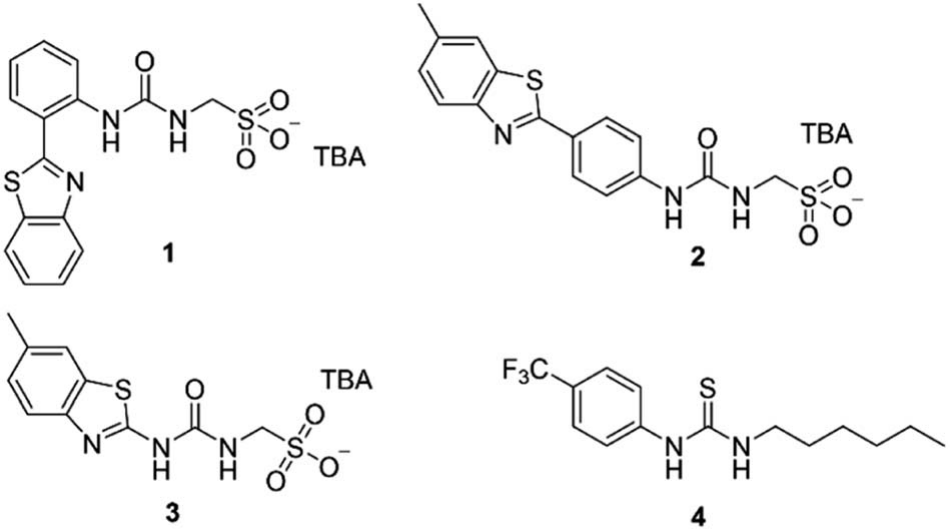
The structures of SSAs 1–3 and anionophore 4 used in this study. TBA is [*n*-tetrabutylammonium]^+^.

In this work we have investigated the cation transport ability of three supramolecular self-associating amphiphiles (SSAs 1–3, Fig. 1), when delivered in a variety of solutions, including 100% water. Key structural components of the SSAs include a sulfonate group to enhance water-solubility and cation binding, whilst the sulfonate and urea moieties are known to promote self-assembly.^17,18^ Recent work has demonstrated that SSAs act as antimicrobial agents and efficacy enhancers for known therapeutics against both bacterial and ovarian cancer cells, with mechanism of action hypothesised to include biological membrane interaction/permeation/disruption events.^19,20^ In addition, SSA 1 has recently been subjected to pre-clinical trials in mice and was found to exhibit a druggable profile, demonstrating target tissue distribution (lung/muscle/liver) and excretion *via* the bloodstream.^21^

## Results and discussion

SSAs 1, 2 and anionophore 4 were synthesised in line with previously published methods.^18,22^ SSA 3 was synthesised through the reaction of tetrabutylammonium (TBA) aminomethanesulfonate with 2-aminobenzothiazole and 1,1′-carbonyldiimidazole (CDI), which gave the desired product as a white solid in a yield of 92% (see Section S1†).

We initially attempted to study ionophoric activity by SSAs 1–3 using the field standard HPTS assay.^23–25^ However, this was not possible due to the intrinsic fluorescence properties of SSA 2, which precluded the detection of changes to the ratiometric HPTS emission. Similarly, a modified K^+^/Na^+^ antiport assay, inspired by recent work from Gale, Sessler and Shin,^2^ was inappropriate for monitoring cation transport events associated with these particular SSA systems (see Section S2.1† for full details). Consequently, a Cl^−^ co-transport or “dual host” assay was employed, inspired by work reported by Moore *et al.*, which allows the cooperative action of two uniport processes to be assessed (Section S2.5†).^26^ Despite the requirement for two uniporters, dual host assays have biological relevance as cell membranes contain native ion channels that synthetic uniporters can couple with and thus enable transport activity within a biological system.^27^

Within the scope of these studies we coupled SSAs 1–3 with the Cl^−^-selective uniporter 4,^22^ using a Cl^−^ selective electrode to monitor the rate of Cl^−^ efflux from 1-palmitoyl-2-oleoyl-*sn*-glycero-3-phosphocholine (POPC) vesicles containing KCl, suspended in an Na_2_SO_4_ buffered system. In the absence of an active counter cation uniporter, Cl^−^-selective uniporter 4 instigates limited/background Cl^−^ efflux. In the presence of an appropriate active cation uniporter, the presence of Cl^−^-selective uniporter 4 enables K^+^/Cl^−^ efflux processes to be initiated, comparatively increasing Cl^−^ efflux rates when compared to the sum of the activity demonstrated by either ionophore independently.

**Fig. 2:**
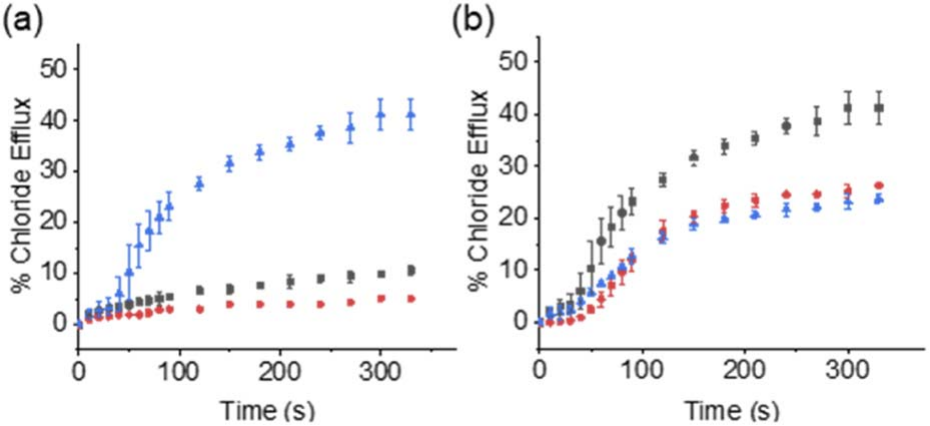
(a) Results from “dual host” assay where DMSO was the delivery solvent, the K^+^ uniporter = SSA 1 and the Cl^−^-selective uniporter = 4. Black squares = 4 only (1 mol% w.r.t. lipid); red circles = 1 only (10 mol% w.r.t. lipid); blue triangles = 1 (10 mol%) + 4 (1 mol%). (b) Comparative results from “dual host” assay where the K^+^ uniporter = SSA 1 and the Cl^−^-selective uniporter = 4. Black squares = SSA 1 (5 mM) delivered in a DMSO solution; red circles = SSA 1 (5 mM) delivered in a 95 : 5 H_2_O/EtOH solution; blue triangles = SSA 1 (5 mM) delivered in a 100% H_2_O solution. Error bars represent a standard deviation for *n* = 3 repeat experiments.

These synthetic vesicle experiments were first conducted using DMSO as the SSA/anionophore delivery solvent. Anionophore 4 (1 mol% w.r.t. lipid) was initially supplied to the vesicle solution, followed by the addition of either DMSO (as a negative control) or SSAs 1–3 (10 mol% w.r.t. lipid). As expected, the addition of anionophore 4 or SSA 1 alone resulted in minimal Cl^−^ efflux (Fig. 2a). However, when SSA 1 and anionophore 4 were combined, significant Cl^−^ efflux was observed. Importantly, the total Cl^−^ efflux was more than the sum of the efflux mediated by 1 and 4 independently. We thus concluded that 1 and 4 function cooperatively to mediate an overall K^+^/Cl^−^ efflux process, and therefore that SSA 1 can function as a K^+^ uniporter. Contrastingly, cooperative transport activity was not observed between 4 and SSA 2 or SSA 3 (Section S2.5†), which instead mediated membrane rupture events.

While it is standard practise to deliver ionophores to vesicles in DMSO, as previously discussed it is also important to assess ionophore deliverability in physiologically relevant solvents, addressing the previously highlighted issues associated with the translation of this technology into the clinic. However, the use of aqueous stock solutions are not standard practise in the field. Previous work has established that the nature and quantity of the solvent can affect the delivery of the transporter to the lipid bilayer and as a result also impact on transporter activity.^28^ In addition, DMSO is known to increase membrane permeability.^29^ While most synthetic transporters have low water solubility, in contrast, SSAs are charged, amphiphilic salts, and are known to dissolve in highly polar, aqueous solvent mixtures, 100% H_2_O (ref. 18) or H_2_O : EtOH (95 : 5).^19^ However, while lower order self-associated SSA species predominate in DMSO solutions, moving into aqueous conditions results in the formation of higher order self-associated species including spherical aggregates and hydrogel fibres,^18,19^ which may affect the concentration of SSA/aggregate type to arrive at the phospholipid vesicle bilayer, and could preclude transport activity. Due to the cation uniport activity identified for SSA 1, and having previously identified this compound to be soluble at the appropriate concentrations within a H_2_O : EtOH solution 19 : 1,^18^ we next moved to establish the solubility and self-associative properties of this same compound in 100% water.

Within 100% H_2_O, the critical micelle concentration (CMC) of SSA 1, was calculated to be 4.13 mM. Increasing the SSA to the desired experimental stock concentration of 5 mM, lead the presence of higher order aggregated species as expected for this class of compounds at a concentration above the CMC (hydrodynamic diameter ≈ 443 nm, see Section S3† for further details). Excitingly, we found that SSA 1 remained active in our K^+^/Cl^−^ co-transport assay when added from either a 5 mM stock solution made up in H_2_O/EtOH (95 : 5) or 100% H_2_O, with some reduction in the observed activity compared to DMSO (Fig. 2b). Hill plot analyses was used to quantify SSA 1 transport efficiency when delivered in a DMSO, H_2_O/EtOH (95 : 5) or H_2_O solution (Table 1). Based on our calculated EC_50_ values, we established that SSA 1 was approximately twice as active when delivered from a DMSO stock solution compared to an aqueous or partially aqueous solution. Despite their modest efficacy, this retention of transport activity using 100% H_2_O as the delivery vehicle represents a step-change in the development of deliverable ionophores, particularly given the established pharmacological profile of this molecule.^21^

**Table 1:** Summary of M^+^ co-transport results for SSA 1 and anionophore 4 (1 mol% w.r.t. lipid) in a range of delivery solvents (5 mM)

Delivery solvent	Co-transport process	EC_50_^a^	*n* b
DMSO	K^+^/Cl^−^	17.0	1.44
H_2_O : EtOH (95 : 5)	K^+^/Cl^−^	29.1	0.95
H_2_O	K^+^/Cl^−^	32.0	0.96
DMSO	Na^+^/Cl^−^	38.5	1.04

a Concentration of SSA 1 required to achieve 50% Cl^−^ efflux after 330 seconds in the presence. Units: mol% with respect to lipid.

b Hill coefficient for the M^+^/Cl^−^ co-transport experiment in the presence of anionophore 4.

We also investigated whether SSA 1 could transport sodium. To do this, we reversed the cation gradient in our co-transport assay to study a Na^+^/Cl^−^ co-transport process (Section S2.8†). As shown in Table 1, SSA 1 retained some activity in this assay with a reduction in the observed EC_50_ value, in line with the Hofmeister classification and the greater hydrophilicity of Na^+^*vs.* K^+^, making it a more challenging ion to transport. We hypothesise that SSA 1 could therefore mediate Na^+^/K^+^ antiport without the need for an additional carrier.

In all cases we found that the observed values for *n*, the Hill coefficient, were close to one. This could be interpreted as evidence to support a unimolecular transport process; however, we note the potential for Hill plot analyses to be complicated by the formation of stable, aggregate species as described by Matile,^30^ since stable supramolecules can be misinterpreted as monomers. Given the extensive data on the dimerisation and aggregation of SSAs under a wide range of conditions,^17–19^ we do not believe that these values offer firm proof of a 1 : 1 SSA: cation transport process.

Finally, we attempted to study the cooperative ion transport by SSA 1 and anionophore 4 using conductance measurements performed across diphytanoylphosphatidylcholine (DPhPC) 10 mol% cholesterol planar phospholipid bilayers using a Port-a-Patch miniaturised patch clamp system (Section S4†). We hoped to gain insight into the transport mechanism of SSA 1. Based on the small molecular, SSA 1 cannot form a unimolecular, membrane-spanning structure; however, given the reported self-assembly capabilities of SSAs,^17,18^ we considered the formation of self-assembled channels a possibility.

**Fig. 3:**
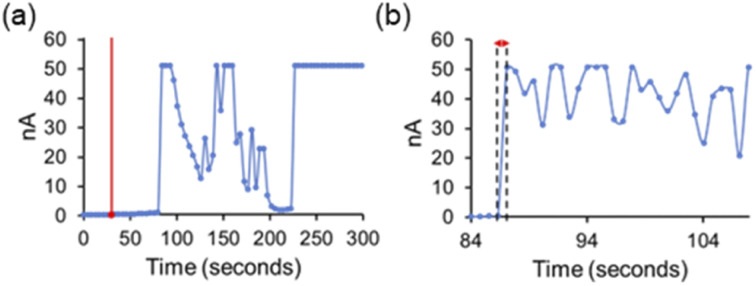
(a) Experimental recording of a DPhPC (10 mol% cholesterol) lipid bilayer at 100 mV after the addition of 4 (0.01 mM) at 0 seconds and SSA 1 (0.375 mM) at 30 seconds. Internal buffer (489 mM KCl, sodium acetate 5 mM, pH 5.5, ionic strength 500 mM) and external buffer (167 mM Na_2_SO_4_ sodium acetate 5 mM, pH 5.5, ionic strength 500 mM). The red line indicates the addition of SSA 1. (b) An equivalent experiment performed with an increased number of data points with respect to time, highlighting the almost instantaneous turn on of change in membrane potential over ≤0.7 seconds.

We found that at lower concentrations (≤0.25 mM), the addition of SSAs 1–3 and 4 alone did not produce an elevated current recording, while higher concentrations of SSA caused bilayer rupture with the SSA acting as a surfactant (see Section S4.3†). However, under specific conditions adding SSA 1 and anionophore 4 together produced an erratic, elevated current flow across the clamped portion of the bilayer, ≈30 seconds after SSA addition, until eventual bilayer rupture was observed ≈ 210 seconds after SSA addition (Fig. 3a). We believe this ‘lag phase’ is due to the time taken for a critical concentration of SSA to accumulate at the planar bilayer before any effects can be observed. Measurements with an increased number of data points with respect to time (Fig. 3b) showed that the observed data are not consistent with the controlled, stepwise, open/close behaviour of single ion channels that is commonly reported for biological and synthetic ion channels, particularly given the magnitude of the current fluctuations (on the nA rather than pA scale). However, we suggest that these concentration dependent mass ion transport events could be representative of SSA 1 operating through a mechanism analogous to the antimicrobial peptide “carpet model” in the presence of anionophore 4.^21,31,32^ Here, peptides accumulate on the bilayer surface until they reach a critical concentration at which membrane disruption can occur.^32^

## Conclusions

In conclusion, we have investigated the ion transport activity of three supramolecular, self-associating anionic amphiphiles with established biological and pharmacological properties. SSA 1 was found to enable both K^+^ and Na^+^ uniport and can be delivered from a 100% water solution. SSA 1 is therefore a novel and water-soluble transport motif that is structurally distinct from traditional transporters which are highly lipophilic. This demonstrates that while high lipophilicity may enhance activity, it is possible to develop active druggable structures which display both water solubility and transport activity. With further development, it may be possible to explore analogues of SSA 1 with the aim of improving activity whilst retaining water-solubility. We therefore believe that charged amphiphilic ionophores represent an exciting prospect for the future development of druggable yet potent synthetic ion transporters that can enable line-of-site to the clinic for this and analogous molecular technologies.

## Author contributions

K. Y., J. E. B.: investigation; validation; writing—original draft, review & editing. L. J. W., K. L. F. H., H. Y. L., Y. L.: investigation; validation; writing—review & editing. J. R. H., C. J. E. H.: conceptualization; funding acquisition; project administration; supervision; writing—original draft, review & editing.

## Conflicts of interest

There are no conflicts to declare.

## Supplementary Material

RA-012-D2RA05314D-s001
