# Supplementary material for: A water-soluble membrane transporter for biologically relevant cations
Source: RSC Adv. 2022 Sep 29;12(43):27877–80. doi: 10.1039/d2ra05314d (PMC9520675; doi:10.1039/d2ra05314d)
Supplement: RA-012-D2RA05314D-s001 [file RA-012-D2RA05314D-s001.pdf]

Electronic Supplementary Material (ESI) for RSC Advances.
This journal is © The Royal Society of Chemistry 2022

Supporting Information

A water-soluble membrane transporter for biologically relevant cations

Kylie Yang,[a,†] Jessica E. Boles,[b,c, †] Lisa J. White,[b] Kira L. F. Hilton,[b] Hin Yuk Lai,[a] Yifan Long,[a] Jennifer R. Hiscock[b]* and Cally J. E. Haynes[a]*

[a] Chemistry Department, UCL, 20 Gordon Street, London WC1H 0AJ, UK. Email: cally.haynes@ucl.ac.uk

[b] School of Chemistry and Forensics, University of Kent, Canterbury, Kent, CT2 7NH, UK. E-mail: J.R.Hiscock@Kent.ac.uk

[c] School of Biosciences, University of Kent, Canterbury, Kent, CT2 7NJ, UK.

[†] These authors contributed equally.

Table of Contents

Supporting Information 1
S1 Synthesis 3
  S1.1 General remarks 3
  S.1.2 Synthetic protocols 3
  S1.3 Molecular characterisation data 4
S2 Ion transport studies 7
  S2.1 Overview of limitations of literature assays in this work 7
  S2.2 General remarks 8
    Preparation of vesicles 8
    Purification of vesicles – fluorescence assays 8
    Purification of vesicles – ion selective electrode assays 8
  S2.3 Fluorescence assays 8
    Method 8
    Data 10
    Analysis 11
  S2.4 K^+ selective electrode assay 11
    Data 12
  S2.5 K^+/ Cl^- co-transport (or “dual host”) assays 13
    Data 13
  S2.6 Deliverability assays 14

Data 14
S2.7 Hill plots 14
Data 15
S2.8 Cation selectivity assay 16
Data: 17
S2.9 pH dependence study 18
Data: 18
S3 Aggregation data 19
S3.1 General remarks 19
S3.2 Experimental methods 19
Tensiometry Studies 19
DLS Studies 19
Zeta Potential Studies 19
S3.3 Results 20
S4 Conductance Measurements 22
S4.1 Materials and methods 22
Solutions and compound preparation 22
Vesicle formation 22
Patch clamp measurements 22
Port-a-Patch chip preparation and data recording procedure: 22
S4.2 Results 24
Controls 24
SSA **1** 25
SSA **2** 30
SSA **3** 35
S4.3 Patch clamp data discussion 40
S5 References 40

S1 Synthesis

S1.1 General remarks

A positive pressure of nitrogen and oven dried glassware were used for all reactions. All solvents and starting materials were purchased from known chemical suppliers or available stores and used without further purification. All NMR spectra were obtained using a Bruker AV2 400 MHz, AVNEO 400 MHz or Avance Neo 500 MHz spectrometer. The data was processed using ACD labs or Topspin software. NMR Chemical shift values are reported in parts per million (ppm) and calibrated to the centre of the residual solvent peak set (s = singlet, br = broad, d = doublet, t = triplet, q = quartet, m = multiplet). The melting point for each compound was measured using a Stuart SMP10 melting point apparatus. High resolution mass spectrometry was performed using a Brucker microTOF-Q mass spectrometer and spectra recorded and processed using Brucker's Compass Data Analysis software. Infrared spectra were obtained using Shimadzu IR-Affinity-1 model Infrared spectrometer. The data was analysed in wavenumbers (cm^{-1}) using IRsolution software.

S.1.2 Synthetic protocols

SSA 1: This compound was synthesized in line with previously published methods.[1] ^{1}H NMR (400 MHz, 298.15 K, DMSO-d6): δ: 10.63 (s, 1H), 8.43 (s, 1H), 8.27 (d, J = 7.79 Hz, 1H), 8.15 (d, J = 7.92 Hz, 1H), 7.85 (d, J = 7.88 Hz, 2H), 7.58 (t, J = 7.58 Hz, 1H), 7.48 (t, J = 11.69, 7.49 Hz, 2H), 7.10 (s, 1H), 3.96 (s, 2H), 3.18 – 3.13 (m, 8H), 1.56 (m, 8H), 1.31 – 1.29 (m, 8H), 0.93 (t, J = 7.34 Hz, 12H).

SSA 2: This compound was synthesized in line with previously published methods.[1] ^{1}H NMR (400MHz, 298K, DMSO-d_6): δ: 9.15 (s, 1H), 7.86 (m, 4H), 7.56 (d, *J* = 8.76 Hz, 2H), 7.32 (dd, *J* = 8.16, 1.2 Hz, 1H), 6.65 (s, 1H), 3.89 (d, *J* = 5.84 Hz, 2H), 3.13 – 3.17 (m, 8H), 2.44 (s, 3H), 1.55 (m, 8H), 1.25 – 1.34 (m, 8H), 0.93 (t, *J* = 7.24 Hz, 12H).

SSA 3: Aminomethansulfonic acid (0.22 g, 2.00 mmol) was dissolved in *n*-tetrabutylammonium (TBA) hydroxide (1N solution in methanol, 2.0 mL) with an additional 15 mL of methanol. This mixture was then taken to dryness to afford TBA aminomethanesulfonate as colourless crystalline solid, assumed yield 100 %.

A mixture of 2-aminobenzothiazole (0.33 g, 2.00 mmol) and 1,1′-carbonyldiimidazole (0.33 g, 2.00 mmol) was heated at reflux for 4 hours in chloroform (20 mL). A solution of TBA aminomethanesulfonate (2.00 mM) in chloroform (10 mL) was then added to the reaction mixture, which was then heated at reflux overnight. The solvent was then removed and the resultant oil dissolved in ethyl acetate (20 mL). The precipitate produced was then removed *via* filtration, washed with ethyl acetate (10 mL) and dried to afford the pure product as a white solid in a yield of 92 % (0.99 g, 1.83 mmol). Melting point: > 200 °C; ^{1}H NMR (400 MHz, 298 K, DMSO-d_6): δ: 10.52 (s, 1H), 7.67 (s, 1H), 7.50 (d, *J* = 8.16 Hz, 2H), 7.11 (d, *J* = 8.20 Hz, 1H); 7.11 (s, 1H); 3.91 (d, *J* = 5.88 Hz, 2H), 3.13 – 3.17 (m, 8H), 2.37 (s, 3H), 1.52 – 1.59 (m, 8H), 1.25 – 1.35 (m, 8H), 0.93 (t, *J* = 7.24 Hz, 12H); ^{13}C{^{1}H} NMR (100 MHz, 298K, DMSO-d_6): δ: 158.7 (ArC), 153.3 (ArC), 147.1 (ArC), 132.0 (ArC), 131.7 (ArC), 127.0 (ArCH), 121.1 (ArCH), 119.5 (ArCH), 57.5 (CH_2), 55.9 (CH_2), 23.1 (CH_2), 21.0 (CH_3), 19.3 (CH_2), 13.6 (CH_3); IR (film): ν (cm^{-1}) = 3345 (NH stretch), 1697 (C=O stretch), 1038 (CN stretch); HRMS for the sulfonate-urea ion ($C_{10}H_{10}N_3O_4S_2^-$) (ESI^-): m/z: act: 300.0133 [M], cal: 300.0118 [M].

Anionophore 4: This compound was synthesized in line with previously published methods.[2] ^{1}H NMR (400 MHz, 298 K, DMSO-d_6): δ: 9.77 (br. s, 1H), 8.03 (br. s, 1H), 7.71 (d, *J* = 8.5, 2H), 7.64 (d, *J* = 8.6, 2H), 3.46 (br. m, 2H), 1.54 (quin., *J* = 6.4, 2H), 1.36 - 1.24 (m, 6H), 0.88 (t, *J* = 6.8 Hz, 3H).

S1.3 Molecular characterisation data

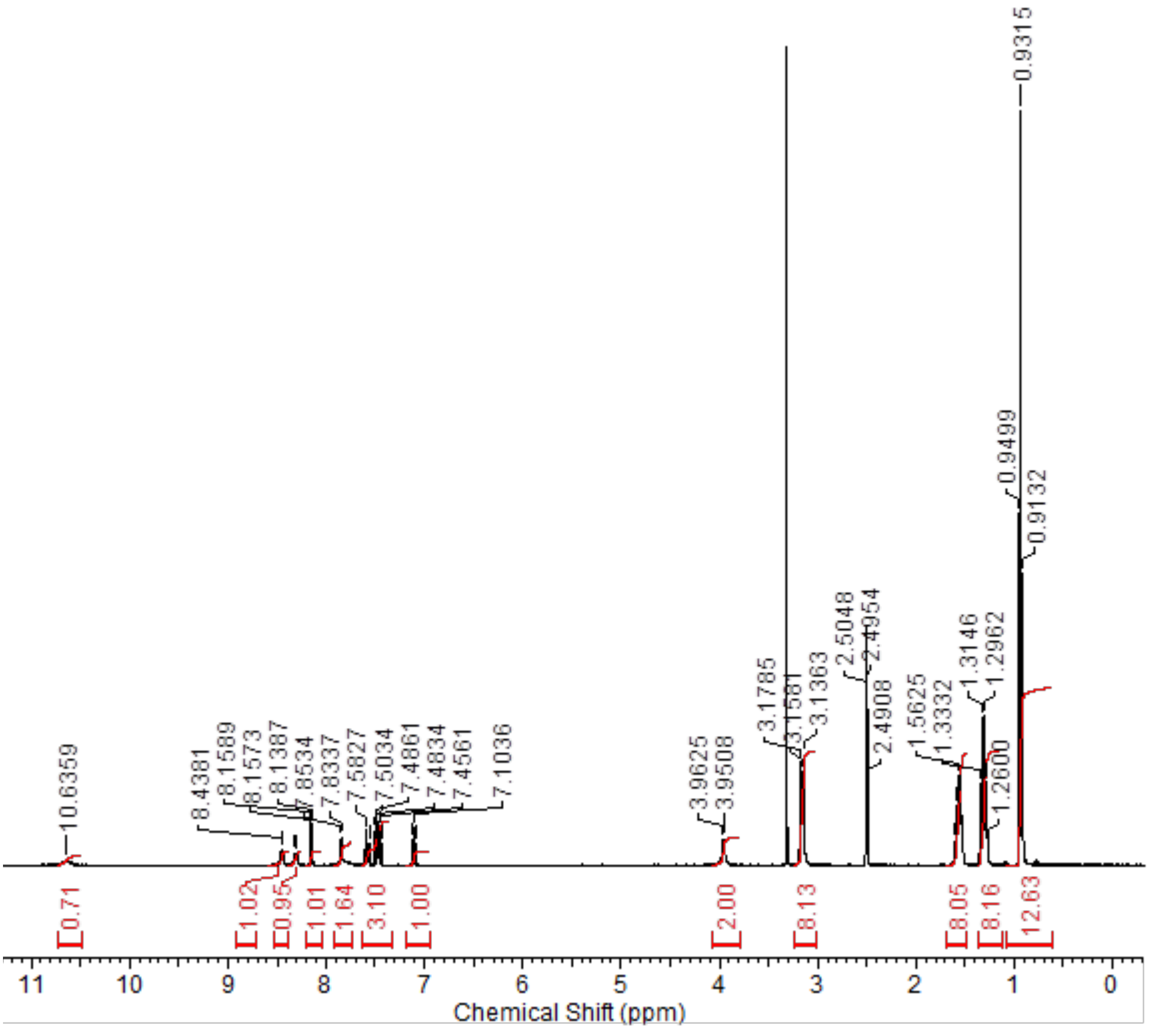

Figure S1 - ^{1}H NMR spectrum (400 MHz, 298 K, DMSO-d_6) of SSA ***1.***

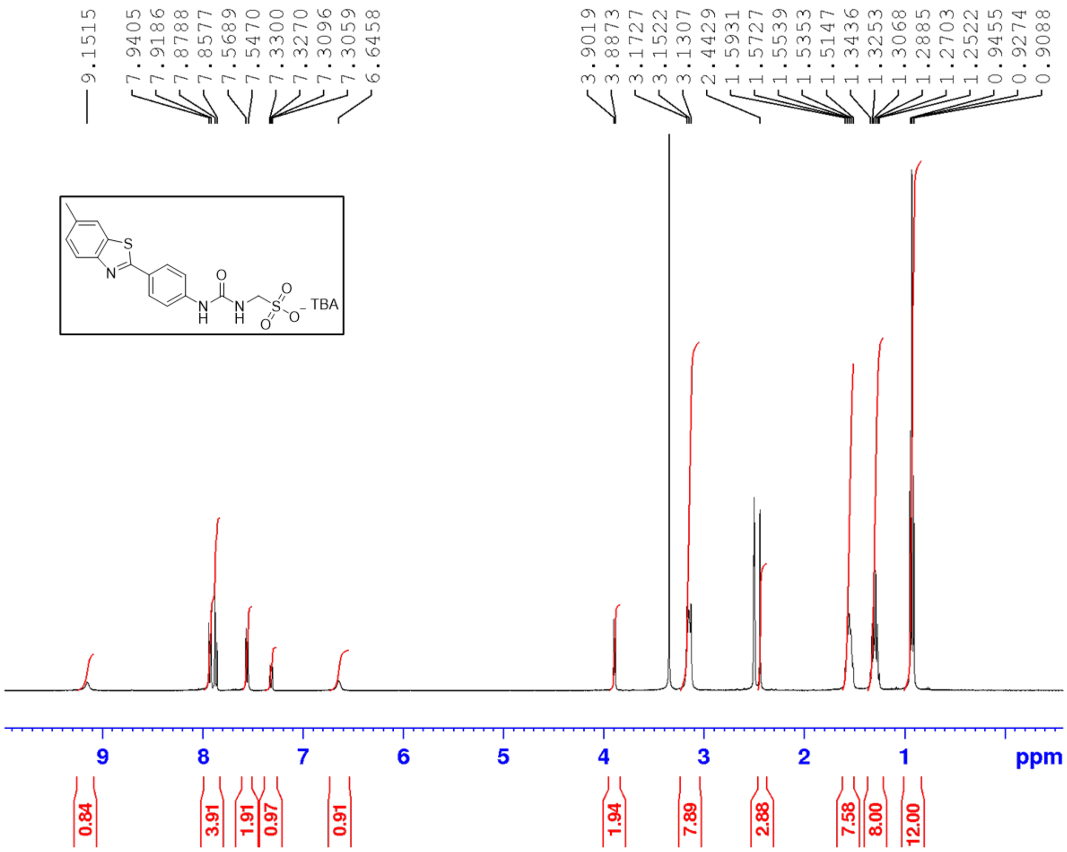

Figure S2 - ^{1}H NMR spectrum (400 MHz, 298 K, DMSO-d$_6$) of SSA ***2****.*

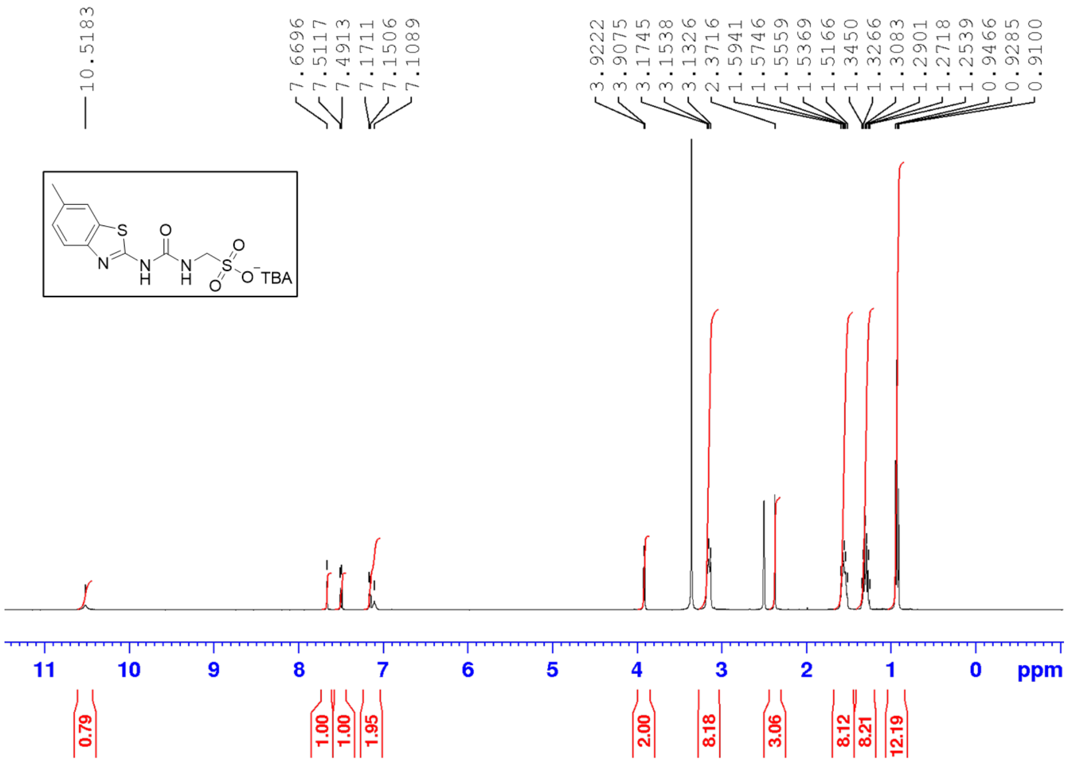

Figure S3 - ^{1}H NMR spectrum (400 MHz, 298 K, DMSO-d$_6$) of SSA ***3****.*

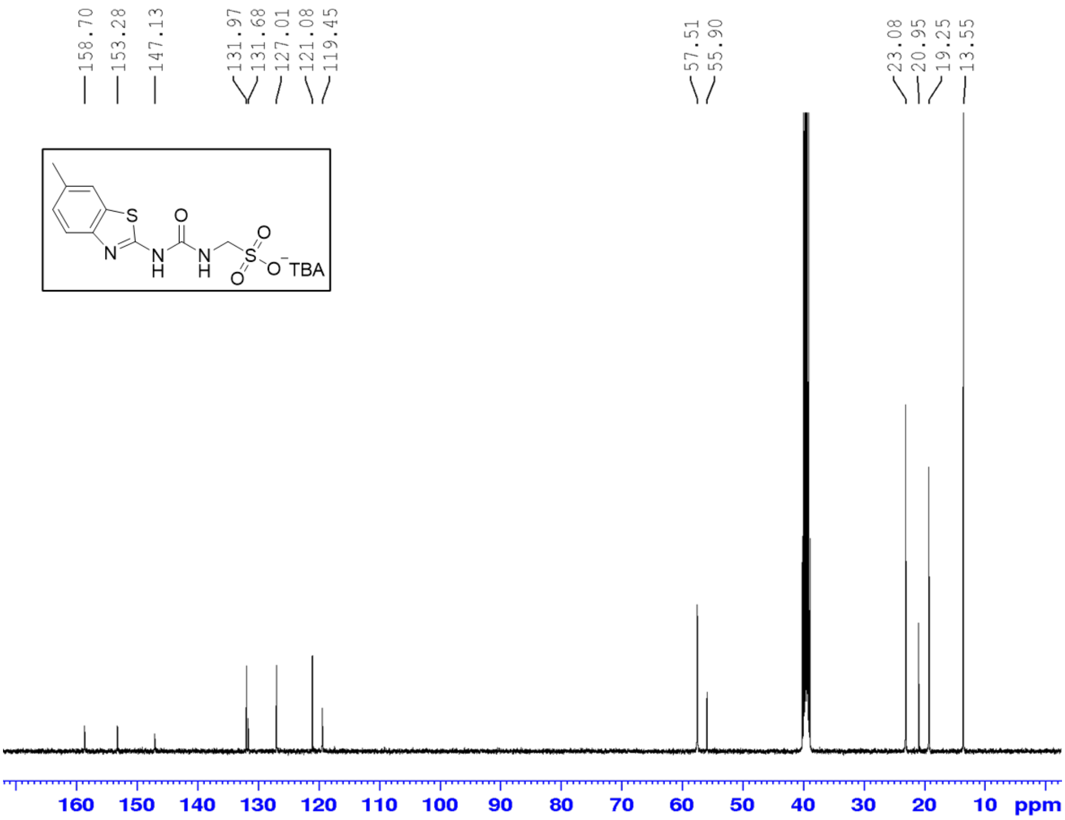

*Figure S4 - $^{13}C\{^{1}H\}$ NMR spectrum (100 MHz, 298 K, DMSO-d_6) of SSA **3**.*

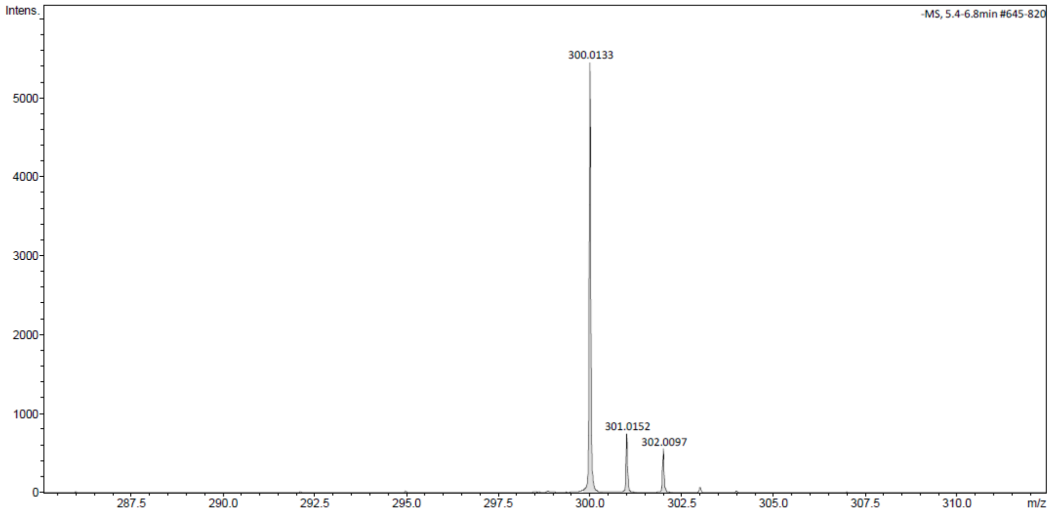

*Figure S5 - A high-resolution mass spectrum (ESI$^-$) obtained for SSA **3** in methanol, m/z [M].*

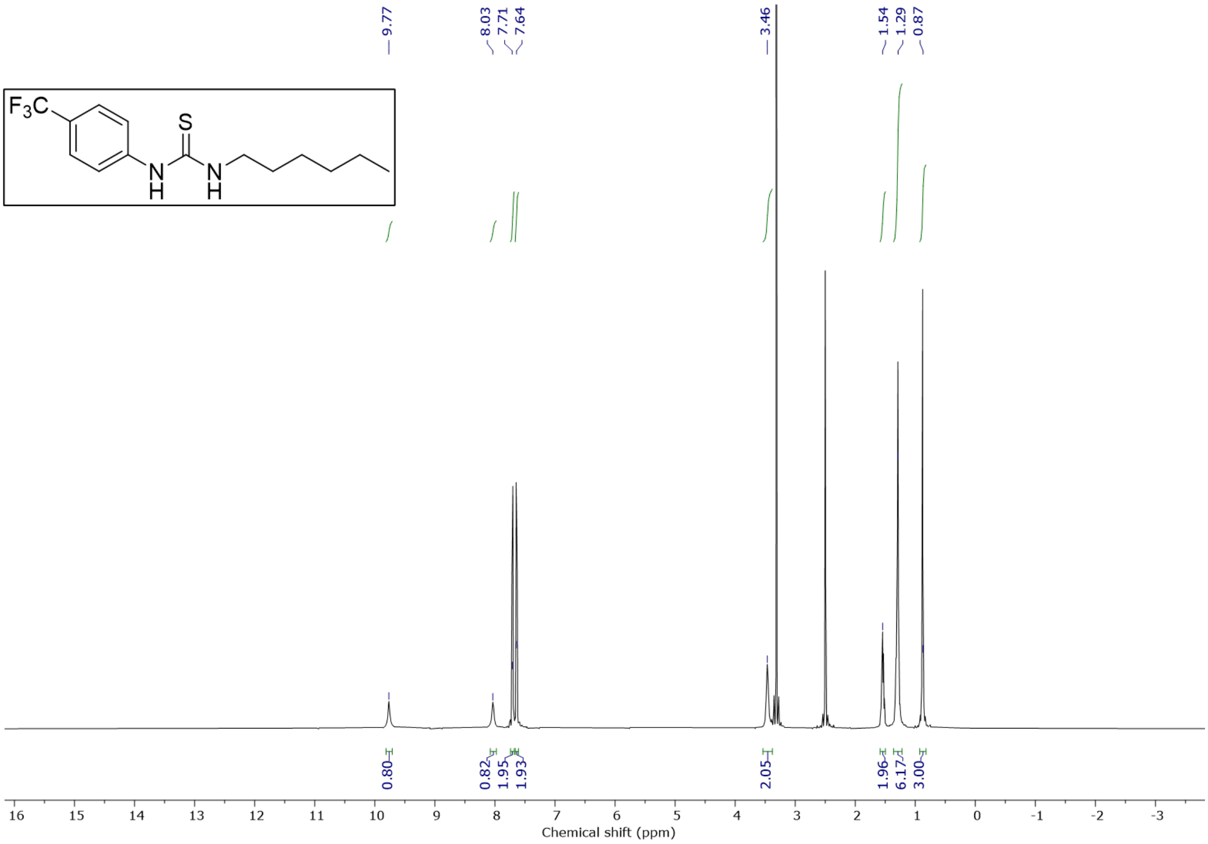

*Figure S6 - ^{1}H NMR spectrum (500 MHz, 298 K, DMSO-d_6) of anionophore **4** in DMSO-d_6.*

S2 Ion transport studies

S2.1 Overview of limitations of literature assays in this work

SSA-mediated cation transport was initially assessed using a field standard HPTS assay.[3] In this assay, a pH sensitive fluorescent dye (HPTS) was encapsulated within synthetic phospholipid vesicles, and M^+/H^+ antiport (or M^+/OH^- symport) assessed through changes in intravesicular pH. The HPTS assay can be adapted to examine M^+ uniport processes in the presence of a proton carrier, such as carbonyl cyanide 3-chlorophenylhydrazone (CCCP). However, the intrinsic fluorescence properties of SSA **2** (Section S2.3) was found to interfere with HPTS emission, thus precluding any further use of this assay. We then explored the use of alternative electrochemical, attempting a modified K+/Na+ antiport assay inspired by recent work from Gale, Sessler and Shin,[4] using a potassium selective electrode to monitor K^+ efflux from vesicles. However, the SSA TBA cation was found to interfere with the potassium electrode, preventing any further use of this method (see Section S2.4).

S2.2 General remarks

Preparation of vesicles

Example procedure:

A stock solution of 1-palmitoyl-2-oleoyl-sn-glycero-3-phosphocholine (POPC, purchased from Merck) was prepared in de-acidified chloroform at a concentration of 1g: 35 mL. The stock solution was stored in a parafilmed, brown glass container at -20 °C.

For a typical transport experiment, 0.5 – 3.0 mL of the POPC solution was transferred to a glass sample vial, and the chloroform was removed under a gentle flow of nitrogen. The resulting lipid film was dried under vacuum for a minimum of 4 hours.

The lipid film was then hydrated with the desired internal buffer solution (a volume equal to the quantity of lipid stock solution used). A small stir bar was added and the sample was vortexed for approximately 1 minute. The lipid sample was then subjected to 9 freeze-thaw cycles. At the end of the freeze-thaw process, the sample was either frozen and stored in the freezer or used on the same day.

After thawing, the lipid was allowed to rest for 30 minutes and then extruded a minimum of 25 times through 200 nm polycarbonate membranes using an Avanti mini-extruder.

Purification of vesicles – fluorescence assays

The vesicles were purified by size exclusion chromatography on Sephadex G50, eluting with the desired external buffer.

Purification of vesicles – ion selective electrode assays

The vesicles were purified by dialysis (10K MWCO) against 1.8 L of the desired external buffer.

S2.3 Fluorescence assays

Method

pH gradient assays using the pH sensitive dye 8-Hydroxypyrene-1,3,6-trisulfonic acid trisodium salt (HPTS) are commonly used in the literature to examine cation transport processes, yet often vary in experimental details including the lipid concentration, buffer compositions and dye concentrations. We chose to base our experiments most closely on conditions reported by Barboiu and co-workers in their study of adaptive transmembrane Na^+ and K^+ carriers.[3]

Using this assay, we intended to assess the ability of SSAs **1–3** to mediate H^+/K^+ antiport, both alone and in the presence of carbonyl cyanide m-chlorophenyl hydrazone (CCCP), a known proton carrier.

Internal buffer solution: Ionic strength = 100 mM (Na_2SO_4), 10 mM sodium phosphate buffer pH 6.4, 10 uM HPTS.

External buffer solution: 100 mM (K_2SO_4), 10 mM sodium phosphate buffer pH 6.4, 10 uM HPTS.

Following purification by size exclusion chromatography, the vesicles were diluted to a known volume using a volumetric flask using the desired external buffer. This lipid stock solution was then further diluted to prepare each sample to a final lipid concentration of 0.18 mM.

To begin an experiment, a cuvette of lipid was placed in the sample holder of a Horiba Fluorolog Fluorometer, maintained with stirring at 25 °C using a Peltier temperature

controller. Data collection was initiated monitoring two emission peaks (λ_{ex} = 403 nm, λ_{em} = 510 nm and λ_{ex} = 460 nm, λ_{em} = 510 nm), corresponding to the acidic and basic forms of the HPTS dye respectively.

At t = 30 s, a DMSO solution of the SSA was added, t = 60 s, a DMSO solution of CCCP (20 μL, 0.1 mol% w.r.t. lipid) was added, and at t = 120 s, a pulse of KOH was added to adjust the external pH to be 7.2 (35 uL of 0.5M KOH, the volume required was confirmed using a pH electrode). After a further 300 s, a solution of Triton-X detergent (31 μL, 10 wt% in water) was added. After a further 120 s, the final reading was taken. Each experiment was performed in triplicate.

After observing unusual results when testing SSA **2**, we also repeated these experiments using vesicles prepared without HPTS in the internal solution to identify the origin of the fluorescence response observed. In each case the raw, unprocessed data is presented in Figures S7–S9.

Data

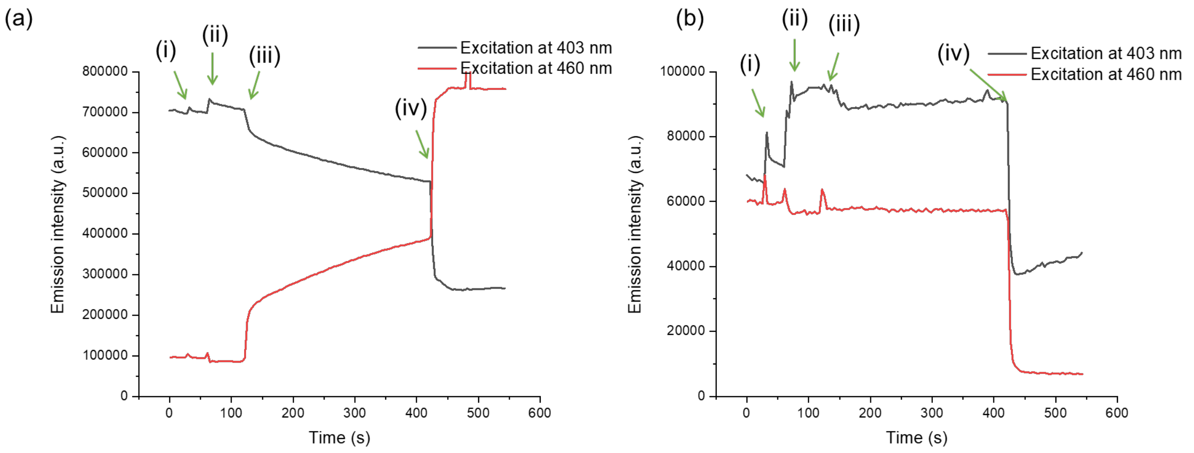

*Figure S7 – Raw data from HPTS assays with SSA **1** (10 mol% w.r.t. lipid) and CCCP (0.1 mol% w.r.t. lipid), showing the emission intensity at 510 nm after irradiation at 403 and 460 nm. (a) Experiment performed using vesicles containing 10 uM HPTS dye; (b) experiment performed using vesicles without HPTS dye. Time points: (i) addition of SSA **1**; (ii) addition of CCCP; (iii) addition of KOH; (iv) addition of detergent to lyse the vesicles.*

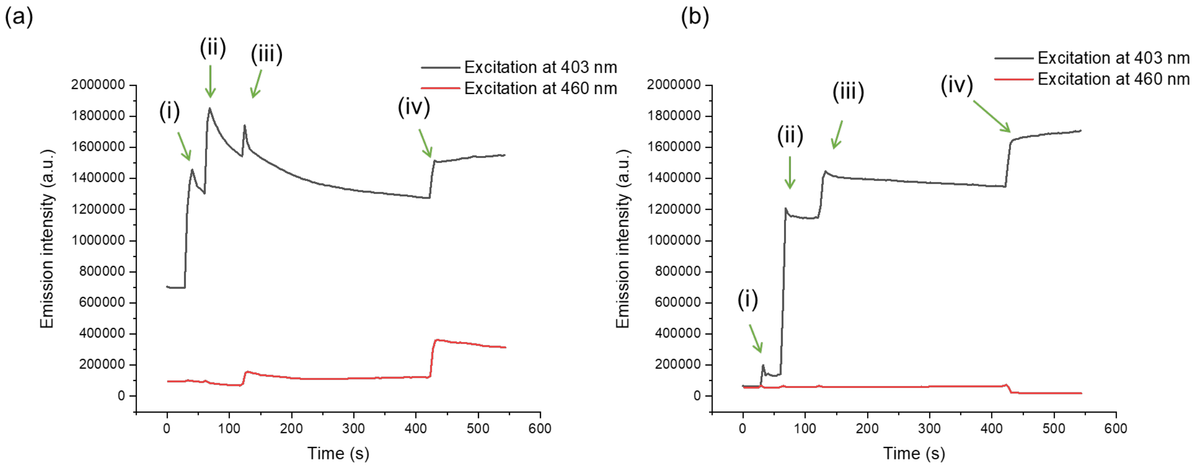

*Figure S8 – Raw data from HPTS assays with SSA **2** (10 mol% w.r.t. lipid) and CCCP (0.1 mol% w.r.t. lipid), showing the emission intensity at 510 nm after irradiation at 403 and 460 nm. (a) Experiment performed using vesicles containing 10 uM HPTS dye; (b) experiment performed using vesicles without HPTS dye. Time points: (i) addition of SSA **1**; (ii) addition of CCCP; (iii) addition of KOH; (iv) addition of detergent to lyse the vesicles.*

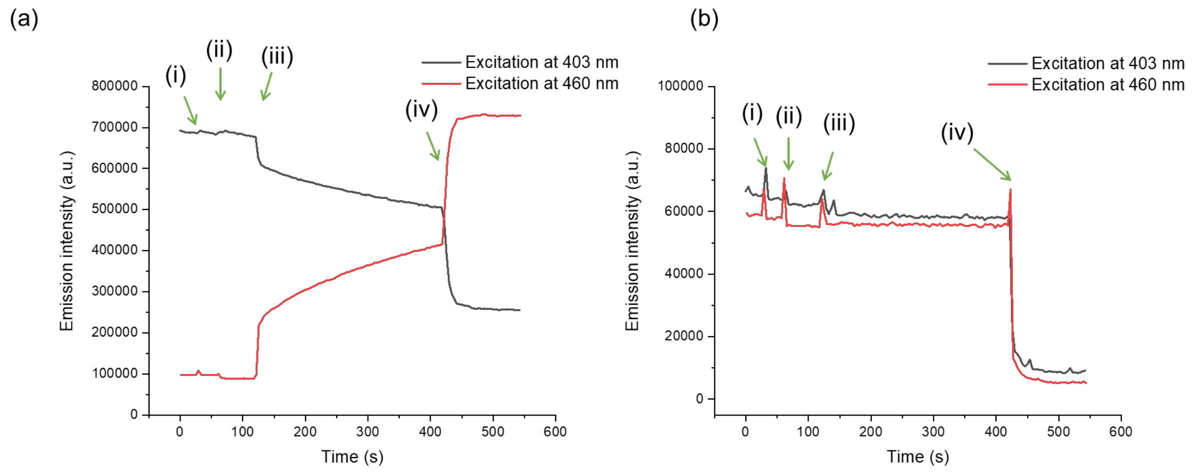

*Figure S9 – Raw data from HPTS assays with SSA **3** (10 mol% w.r.t. lipid) and CCCP (0.1 mol% w.r.t. lipid), showing the emission intensity at 510 nm after irradiation at 403 and 460 nm. (a) Experiment performed using vesicles containing 10 uM HPTS dye; (b) experiment performed using vesicles without HPTS dye. Time points: (i) addition of SSA **1**; (ii) addition of CCCP; (iii) addition of KOH; (iv) addition of detergent to lyse the vesicles.*

<u>Analysis</u>

The results indicated that SSA **2** caused a large increase in emission intensity (ex 403 nm, em 510 nm) in both assays after the addition of CCCP (Figure S8). We also observed a small increase in emission intensity (ex 403 nm, em 510 nm) after the addition of SSA **1** and CCCP in the experiment without HPTS (Figure S7(b)), although the magnitude of the increase was smaller and less significant by comparison to the fluorescence of the HPTS dye. These increases in fluorescence emission, particularly with SSA **2**, meant that monitoring the fluorescence response of the HPTS dye to any potential ion transport processes was impractical. We also noted that the residual fluorescence of the HPTS after lysing the vesicles was different in each case, which may provide some evidence of an interaction between the HPTS and the SSAs. We concluded that this assay was therefore unsuitable to yield a reliable and comparative transport dataset for SSAs **1**–**3** and sought to investigate alternative assays.

S2.4 K^+ selective electrode assay

We modelled our K^+ selective electrode assay on a protocol reported by Gale, Sessler and Shin.[4]

Internal buffer solution: Ionic strength = 300 mM (K_2SO_4), 5 mM sodium phosphate buffer pH 7.2.

External buffer solution: Ionic strength = 300 mM (Na_2SO_4), 5 mM sodium phosphate buffer pH 7.2.

Following purification by dialysis, the vesicles were diluted to a known volume using a volumetric flask and each sample was prepared at a final lipid concentration of 1 mM.

A vial of 1 mM lipid was stirred rapidly, and the external K^+ concentration was monitored using an Oakton by Cole-Parmer® Combination Potassium Ion-Selective Electrode. To start the experiment, a solution of the SSA in DMSO (5 mM stock solution) was added and

changes in the mV reading of the electrode were recorded. After 300 s, a solution of Triton-X detergent (20 μL, 10 wt% in water) was added and a final K^+ concentration was taken after 420 s. This final reading was used to calibrate 100% K^+ efflux. For comparison, we also tested a solution of *n*-tetrabutylammonium nitrate in DMSO (5 mM), and established that the *n*-tetrabutylammonium cation is a likely interferant for the K^+ selective electrode.

Data

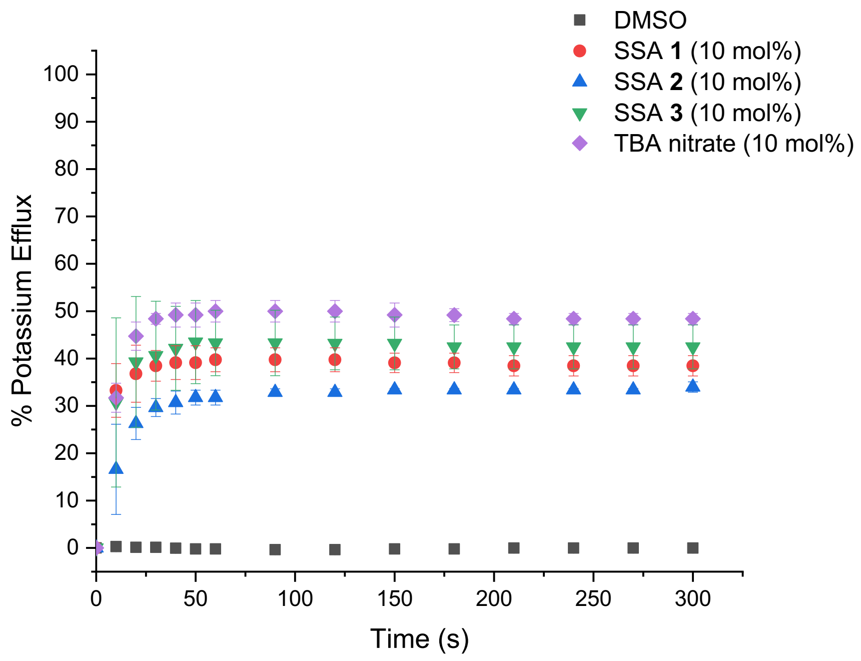

*Figure S10 - Results from the K^+ selective electrode assay showing apparent changes in external K^+ concentration upon addition of 10 mol% of SSAs **1-3** and TBA nitrate from a 5 mM DMSO stock solution.*

S2.5 K^+/ Cl^- co-transport (or "dual host") assays

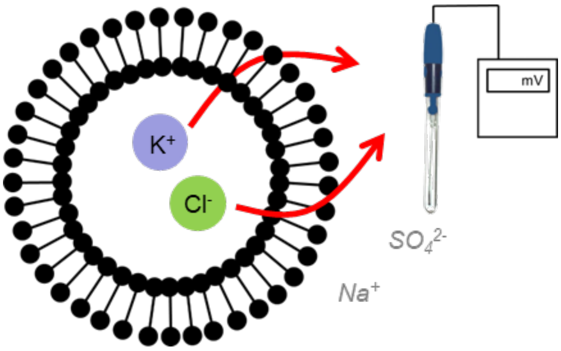

*Figure S11 – Schematic representation of the K^+/ Cl^- co-transport assay used in this work. Here the Cl^- anion efflux is mediated by anionophore **4** and the K^+ cation efflux is proposed to be mediated by the SSA. Cl^- anion efflux is detected using a chloride selective electrode. Counterions shown in grey are thought not to influence the overall transport process.*

Internal buffer solution: Ionic strength = 500 mM (KCl), 5 mM sodium phosphate buffer pH 7.2.

External buffer solution: Ionic strength = 500 mM (Na_2SO_4), 5 mM sodium phosphate buffer pH 7.2.

Following purification by dialysis, the vesicles were diluted to a known volume using a volumetric flask and each sample was prepared at a final lipid concentration of 1 mM.

A vial of 1 mM lipid was stirred rapidly, and the external Cl^- concentration was monitored using an Oakton by Cole-Parmer® Combination Chloride Ion-Selective Electrode. To start the experiment, a solution of anionophore **4** (1 mol% w.r.t. lipid) was added from a 10 mM DMSO stock solution. At t = 30 s, the SSA in DMSO (5 mM stock solution) was added and changes in the mV reading of the electrode were recorded. After 330 s, a solution of Triton-X detergent (20 μL, 10 wt% in water) was added and a final Cl^- concentration was taken after 450 s. This final reading was used to calibrate 100% Cl^- efflux.

Data

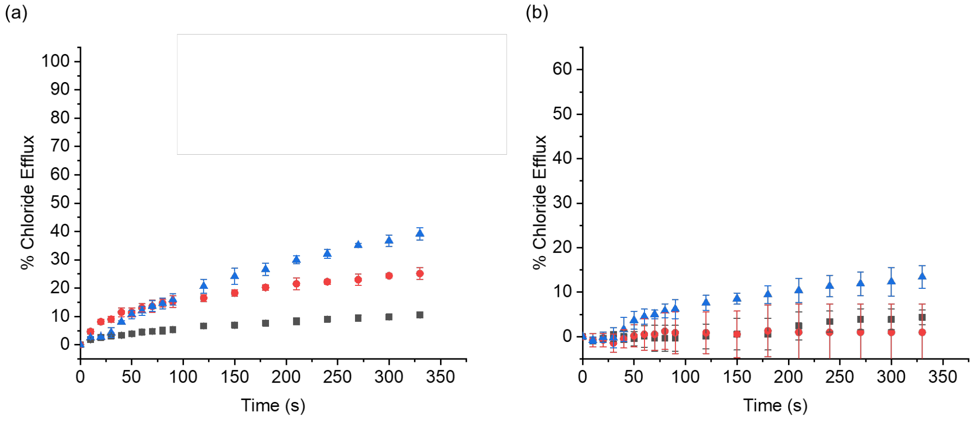

*Figure S12 - Results from of the K^+/Cl^- co-transport assay using (a) SSA **2** and (b) SSA **3**, both in combination with anionophore **4**. Black squares = **4** only (1 mol% w.r.t. lipid); red circles = SSA only (10 mol% w.r.t. lipid); blue triangles = SSA (10 mol%) + **4** (1 mol%). Results for SSA **1** are shown in the main paper, figure 2(a).*

In the K^+/Cl^- co-transport screening experiments using SSAs **2** and **3**, we found that each of these molecules was able to mediate a small amount of chloride efflux independently of the addition of anionophore **4** (Figures S12(a) and 2(b)). However, the total chloride efflux observed when adding these SSAs in combination with anionophore **4** was not significantly larger than the sum of the efflux mediated by the SSA and **4** independently. We hypothesise, that due to the amphiphilic nature of SSAs, that these observations maybe attributed to the SSA surfactant which result in small amounts of vesicle lysis at higher concentrations (see also Section S4). This is in contrast to the behaviour of SSA **1,** which does not mediate any chloride leakage in the absence of anionophore **4** (See Figure 1a in the main text) and has been reported not to mediate significant calcein leakage from POPC vesicles at the concentrations used in this work.[5]

S2.6 Deliverability assays

The experiments were run as described in Section S2.4, except SSA **1** was made as a fresh stock solution in 95:5 H_2O/ EtOH (5 mM) or H_2O (5 mM). Solutions of SSA **1** in H_2O were prepared by heating a suspension of the SSA in H_2O to 70 °C with a thermomixer. Before each addition, the Eppendorf was inverted.

<u>Data</u>

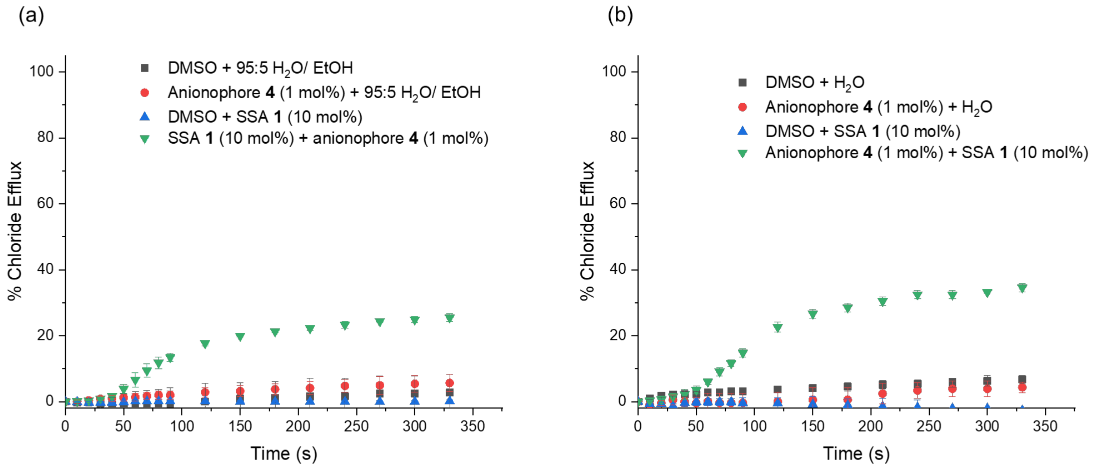

*Figure S13 – Full screening results from the K^+/Cl^- co-transport assay using (a) SSA **1** as a 5 mM stock solution in 95:5 H_2O/ EtOH, and (b) SSA **1** as a 5 mM stock solution in 100% H_2O.*

S2.7 Hill plots

Using the conditions described in Section S2.4, we performed Hill plot analyses in order to quantify the observed transport. SSA **1** was tested at a variety of concentrations in combination with 1 mol% of anionophore **4**. The chloride efflux after 330 s was plotted against the transporter loading, and the data was fitted to a Hill equation to derive values of k (the $EC_{50,\,330\,s}$ value) and n (the Hill coefficient). This analysis was performed using 5 mM stock solutions of SSA **1** prepared in DMSO, 95:5 H_2O/ EtOH and 100% H_2O.

Data

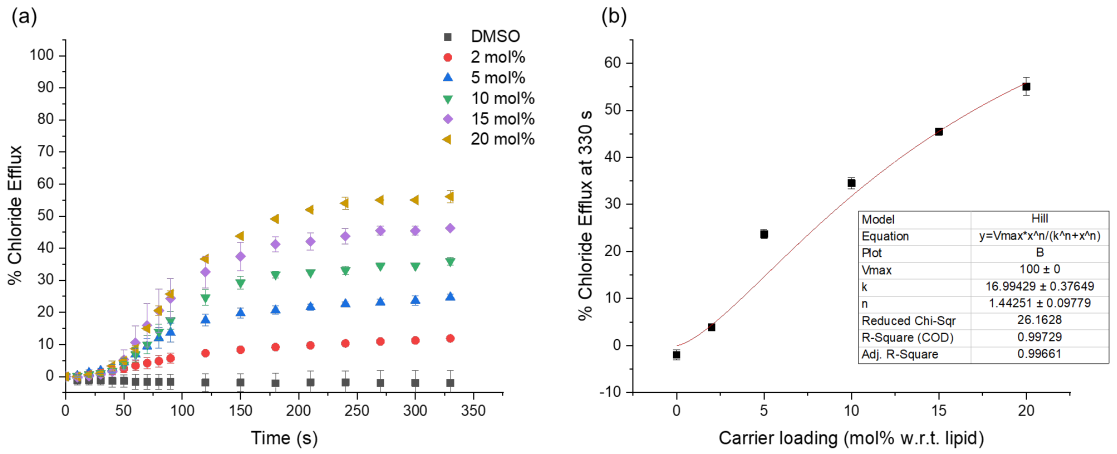

*Figure S14 – Hill plot analysis for SSA **1** (5 mM DMSO stock solution) in the presence of 1 mol% anionophore **4**: (a) raw data showing the response to increasing concentrations of SSA **1**; (b) Hill analysis showing calculated parameters k ($EC_{50,\ 330\ s}$) and n (Hill coefficient).*

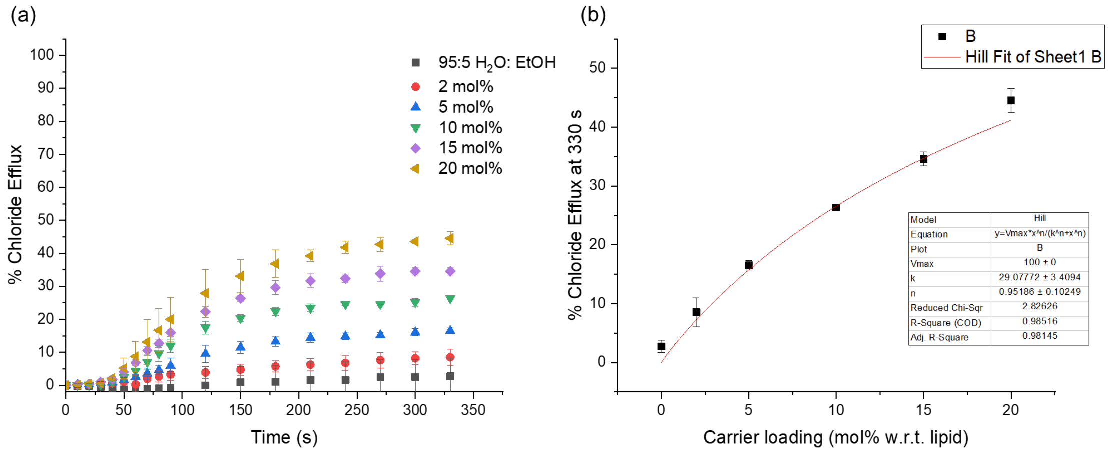

*Figure S15 – Hill plot analysis for SSA **1** (5 mM 95:5 H_2O: EtOH stock solution) in the presence of 1 mol% anionophore **4**: (a) raw data showing the response to increasing concentrations of SSA **1**; (b) Hill analysis showing calculated parameters k ($EC_{50,\ 330\ s}$) and n (Hill coefficient).*

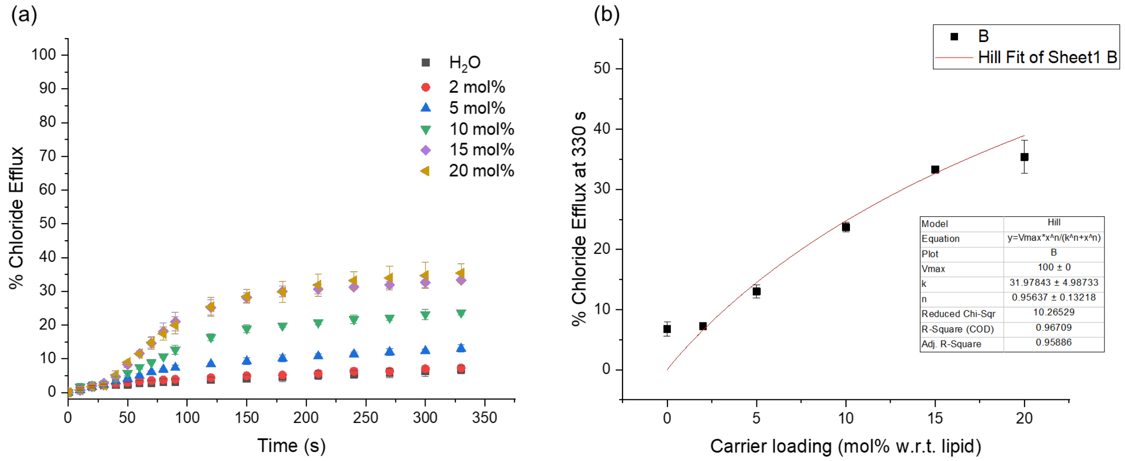

*Figure S16 – Hill plot analysis for SSA **1** (5 mM stock solution in H_2O) in the presence of 1 mol% anionophore **4**: (a) raw data showing the response to increasing concentrations of SSA **1**; (b) Hill analysis showing calculated parameters k ($EC_{50,\ 330\ s}$) and n (Hill coefficient).*

S2.8 Cation selectivity assay

The experiment was run as described in Section S2.4 and S2.6, except the internal and external buffers were modified as follows:

Internal buffer solution: Ionic strength = 500 mM (NaCl), 5 mM sodium phosphate buffer pH 7.2.

External buffer solution: Ionic strength = 500 mM (K_2SO_4), 5 mM sodium phosphate buffer pH 7.2.

<u>Data:</u>

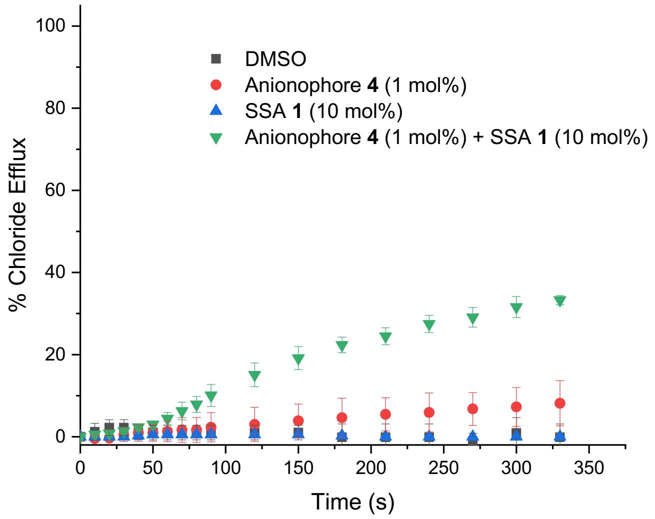

*Figure S17 – Full screening results from the Na$^+$/Cl$^-$ co-transport assay using SSA **1** and anionophore **4**.*

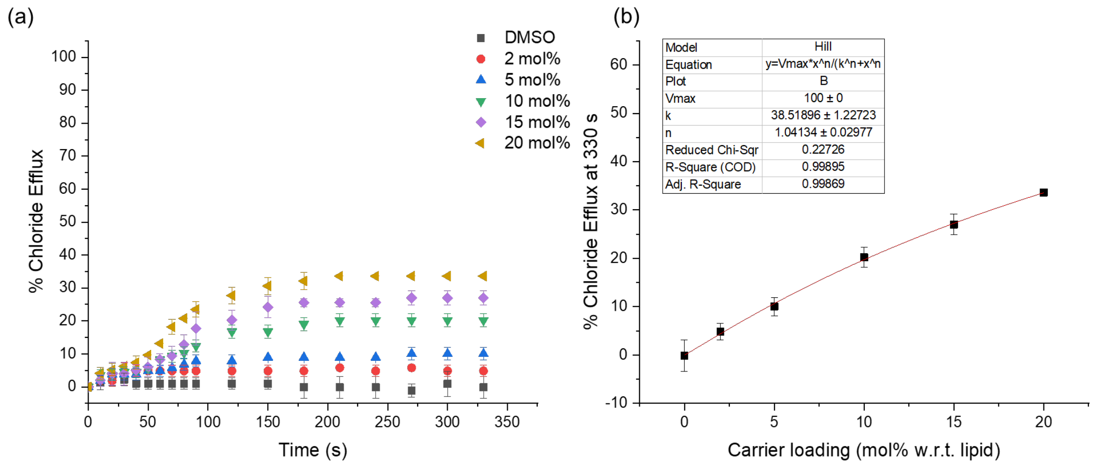

*Figure S18 - Hill plot analysis for SSA **1** (5 mM DMSO stock solution) mediating Na$^+$/ Cl$^-$ co-transport in the presence of 1 mol% anionophore **4**: (a) raw data showing the response to increasing concentrations of SSA **1**; (b) Hill analysis showing calculated parameters k ($EC_{50,\ 330\ s}$) and n (Hill coefficient). Fitting was performed using unweighted values.*

S2.9 pH dependence study

Since the conductance measurements described in section S3 needed to be performed using buffers at pH 5.5 (in order to obtain a stable planar bilayer clamp), we wanted to verify whether SSA **1** remained similarly active as a transporter at pH 5.5.

The experiment was run as described in Section S2.4, except the internal and external buffers were modified as follows:

Internal buffer solution: Ionic strength = 500 mM (KCl), 5 mM sodium citrate buffer pH 5.5.

External buffer solution: Ionic strength = 500 mM (Na_2SO_4), 5 mM sodium citrate buffer pH 5.5.

Data:

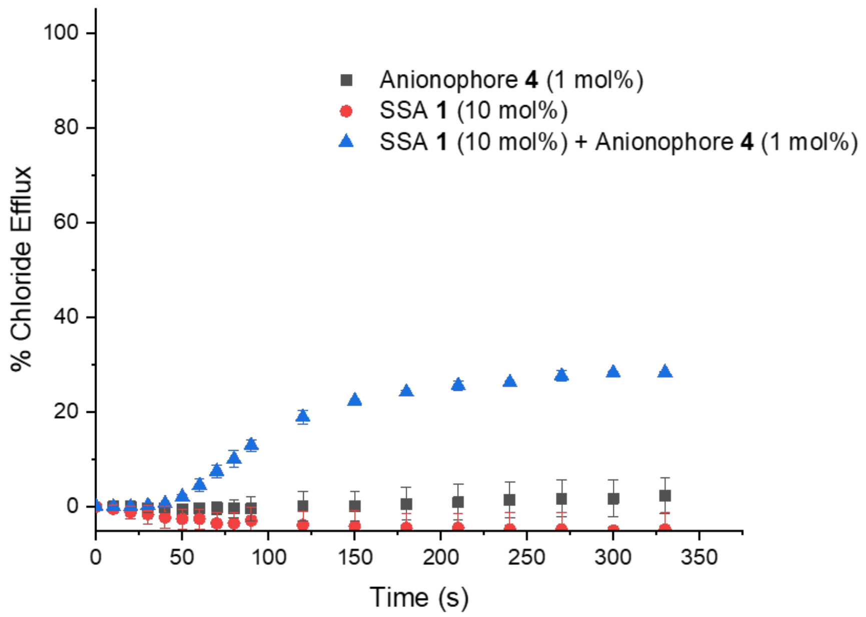

*Figure S19 – data from the pH dependence study: K^+/Cl^- co-transport experiment carried out at pH 5.5 (internal and external solutions) using SSA **1** (10 mol% w.r.t. lipid, 5 mM DMSO stock solution) and anionophore **4** (1 mol% w.r.t. lipid).*

S3 Aggregation data

The self-association properties of SSA **1** have been previously reported in DMSO and H_2O/ EtOH 95:5.[1] After observing the solubility of SSA **1** in 100% H_2O, we carried out full characterisation of the self-association properties under these solvent conditions as follows.

S3.1 General remarks

Tensiometry measurements were undertaken using the Biolin Scientific Theta Attension optical tensiometer. The data was processed using Biolin OneAttension software. A Hamilton (309) syringe was used for the measurements. DLS and Zeta Potential studies were carried out using Anton Paar Litesizer™ 500 and processed using KalliopeTM Professional.

S3.2 Experimental methods

Tensiometry Studies

All the samples were prepared in deionised water. All samples underwent an annealing process in which the various solutions were heated to approximately 40 °C before being allowed to cool to room temperature, allowing each sample to reach a thermodynamic minimum. All samples were prepared through serial dilution of the most concentrated sample. Three surface tension measurements were obtained for each sample at a given concentration, using the pendant drop method. The average values were then used to calculate the critical micelle concentration (CMC).

DLS Studies

All solvents used were filtered to remove any particulates that may interfere with the results obtained. All samples underwent an annealing process, in which they were heated to 40 °C before being allowed to cool to 25 °C. The final intensity weighted particle size distribution given is an average of 10 repeated experiments conducted at 25 °C.

Zeta Potential Studies

All solvents used were filtered to remove any particulates that may interfere with the results obtained. All samples underwent an annealing process in which the various solutions were heated to approximately 40 °C before cooling to room temperature, allowing each sample to reach a thermodynamic minimum. The final zeta potential value given is an average of 10 repeated experiments conducted at 25 °C.

S3.3 Results

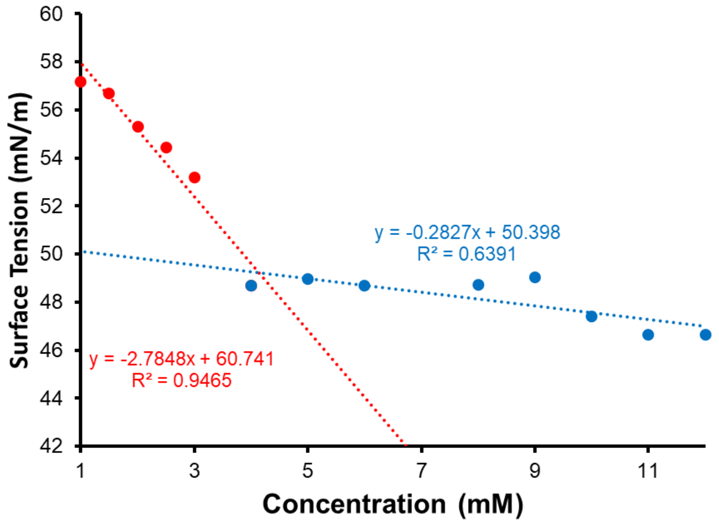

*Figure S20 - Calculation of CMC (4.13 mM) for SSA **1** in deionised water using surface tension measurements.*

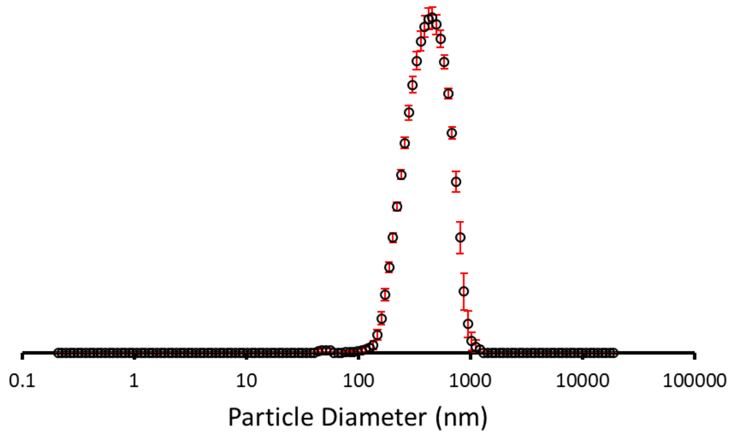

*Figure S21 - The average intensity particle size distribution calculated from 10 repeat experiments for SSA **1** (5.0 mM) in deionised water at 298 K.*

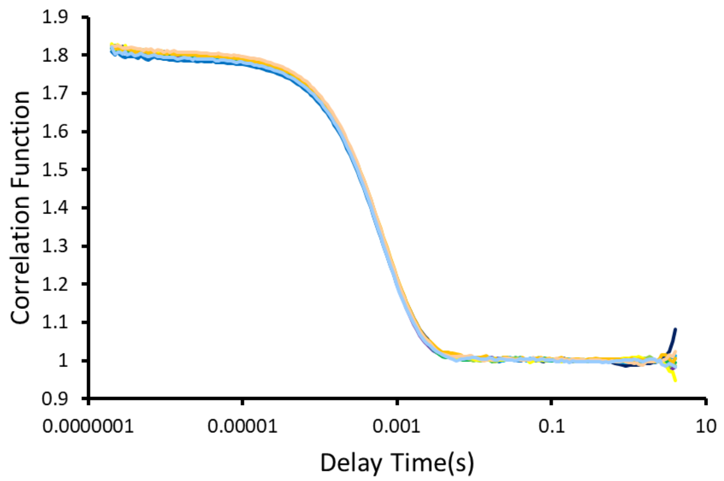

*Figure S22 - Correlation function data obtained from DLS studies (n = 10) of SSA **1** (5.0 mM) in deionised water at 298 K.*

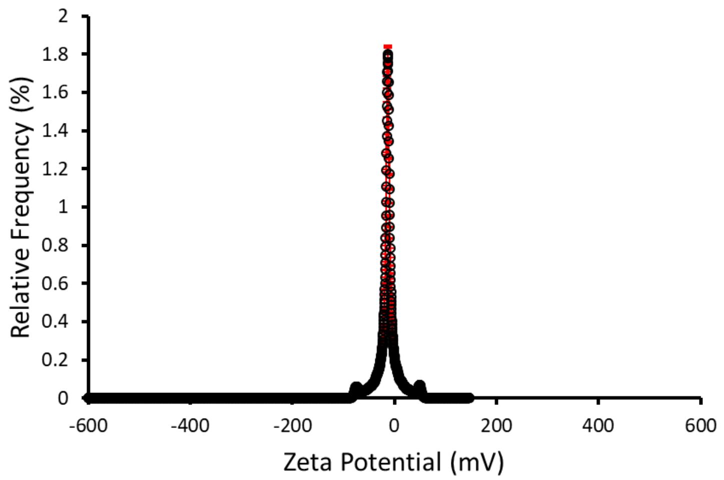

*Figure S23 - The average zeta potential distribution calculated from 10 repeat experiments for SSA **1** (5.0 mM) in deionised water at 298 K. Average measurement value **-**14.8 mV.*

S4 Conductance Measurements

S4.1 Materials and methods

Solutions and compound preparation

All solvents and starting materials were purchased from known chemical suppliers. Buffer A (489 mM KCl, acetate 5 mM, pH 5.5, ionic strength 500 mM) and Buffer B (167 mM Na_2SO_4, acetate 5 mM, pH 5.5, ionic strength 500 mM). Stock solutions of SSAs and AT were prepared in DMSO.

Vesicle formation

Giant unilamellar Vesicles (GUVS) were prepared using the electroformation method using the Nanion VesiclePrepPro setup. A solution of 1,2-diphytanoyl-sn-glycero-3-phosphocholine (DPhPC) (10 mM) with 10 % cholesterol in chloroform (20 μL) was deposited on the ITO-coated glass surface to create a dehydrated thin lipid film. This lipid/ cholesterol mixture was selected as it allowed for the formation of a stable bilayer under these experimental conditions. A greased O-ring was filled with sucrose (200 mM, 275 μL) and sealed with a second ITO-slide. AC current (3 V, 5 Hz) was then applied. After 2 h the voltage and the frequency were reduced in steps, first to 1.6 V/1 Hz, after 30 min and held for 1 h and to the final values 0V/0 Hz after 30 mins.

Patch clamp measurements

Patch clamp experiments were performed with the Port-a-Patch (Nanion Technologies), using borosilicate glass chips with an aperture diameter of approximately 1 μm. The GUVS were positioned into the aperture in the chip by application of negative pressure (-30 mbar), with symmetrical buffer A solutions. The external buffer was exchanged with buffer B. A single GUV forms a planar bilayer upon contact with the glass surface of the chip. The holding potential was set to 50 or 100 mV. AT (0.01 mM) was added at t = 0 s and the SSA (0.1 - 1 mM) was subsequently added at t = 30 s. The sweep interval was set to 1 s, and the protocol was run for a total of 5 mins in triplicate.

Port-a-Patch chip preparation and data recording procedure:

1. 5 μL of buffer A is applied to the underside of patch clamp disposable chip (resistance = 3.5 mΩ).
2. 15 μL of buffer A is applied to the top of patch clamp disposable chip.
3. 5 μL of the GUV stock solution is then added to the top of the chip (lipid concentration 10 mM – for preparation see separate section relating to this within this document).
4. The planar bilayer is then formed through application of negative pressure (-30 mbar). When the live readout shows the planar bilayer has formed, the negative pressure is removed.
5. Then the buffer A:vesicle stock solution 3:1, which sits onto of the chip, is exchanged for buffer B through 4 x 15 μL solution exchanges.
6. Measurements are then initiated through addition of anion transporter solution (5 μL) followed by SSA stock solution after 30 seconds (5 μL) as appropriate.

7. 15 µL of the mixture created in step 6 is then added back to the top of the chip and the time point recorded. This process is undertaken during continuous measurement collection. Where the SSA and anion transporter were present in the same experiment, the anion transporter was added at time = 0 seconds, and the SSA was added through the standard addition procedures given in step 6.

Buffer A = KCl (489 mM), Na acetate (5 mM), pH 5.5, ionic strength 500 mM.

Buffer B = Na_2SO_4 (167 mM), Na acetate (5 mM), pH 5.5, ionic strength 500 mM.

Anion transporter (AT) solution = 2.5 mL of buffer B and 5 µL of **4** (10 mM) in DMSO.

SSA stock solution = 2.5 mL of buffer B and 5 µL of SSA in DMSO.

The limit of detection of the Port-a-Patch instrument is 50 nA under these experimental conditions. A second live trace informs of any bilayer rupture events which take place during the experiment, reporting the loss of membrane resistance. These events are recorded as a constant 50 nA readout by the instrument and represent the current thought the electrolyte in the absence of membrane.

S4.2 Results

Controls

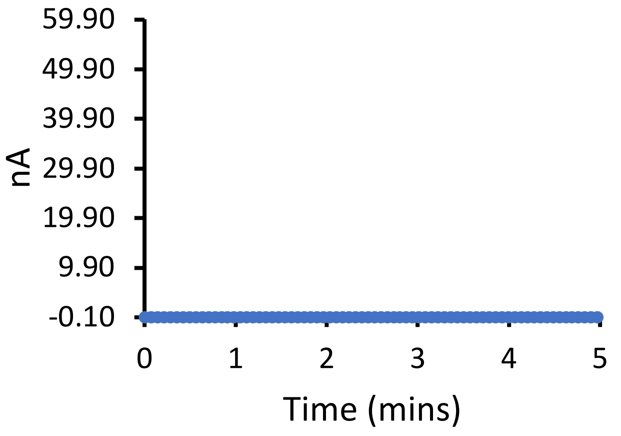

Figure S24 - Experimental recording 1 of a DPhPC bilayer at 100 mV, addition of DMSO control.

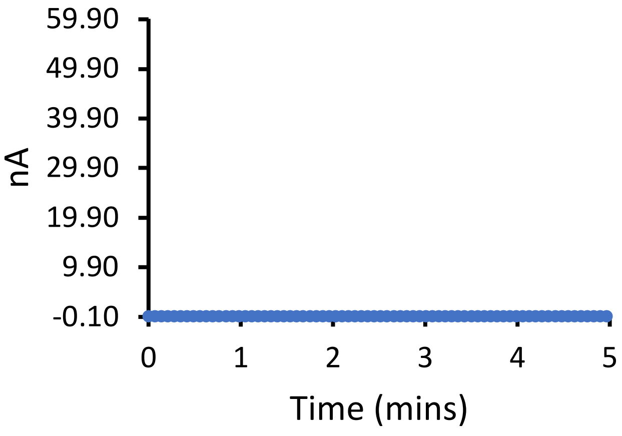

Figure S25 - Experimental recording 2 of a DPhPC bilayer at 100 mV,addition of DMSO control.

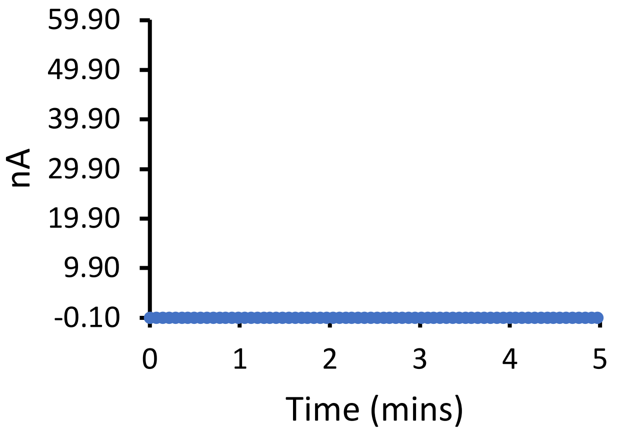

Figure S26 - Experimental recording 3 of a DPhPC bilayer at 100 mV, addition of DMSO control.

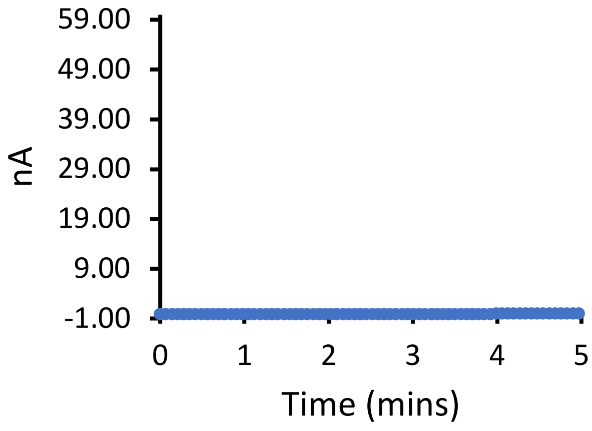

*Figure S27 - Experimental recording 1 of a DPhPC bilayer at 100 mV,addition of **4.***

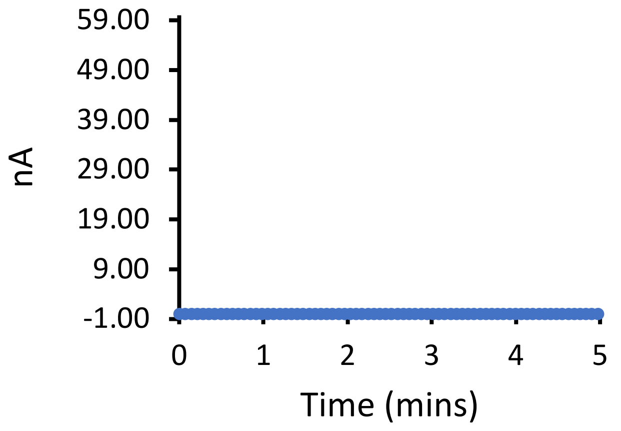

*Figure S28 - Experimental recording 2 of a DPhPC bilayer at 100 mV, addition of **4.***

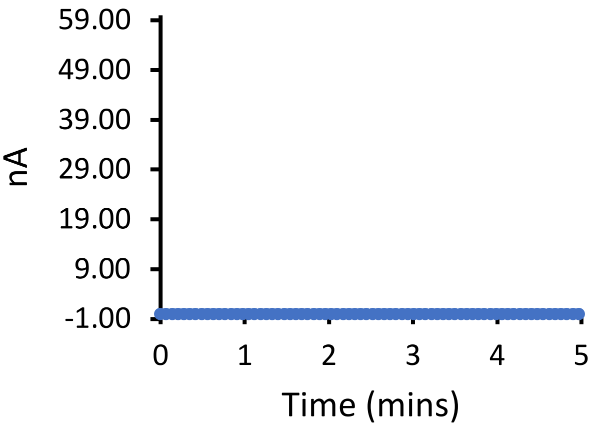

*Figure S29 - Experimental recording 3 of a DPhPC bilayer at 100 mV, addition of **4.***

SSA **1**

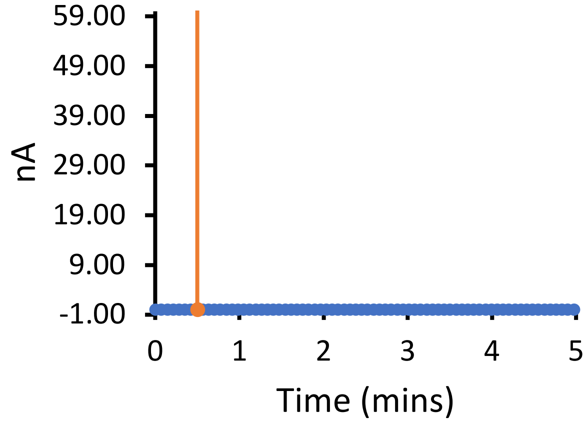

*Figure S30 – Experimental recording 1 of a DPhPC bilayer at 100 mV, addition of **1** (0.10 mM) after 30 seconds indicated by the orange line.*

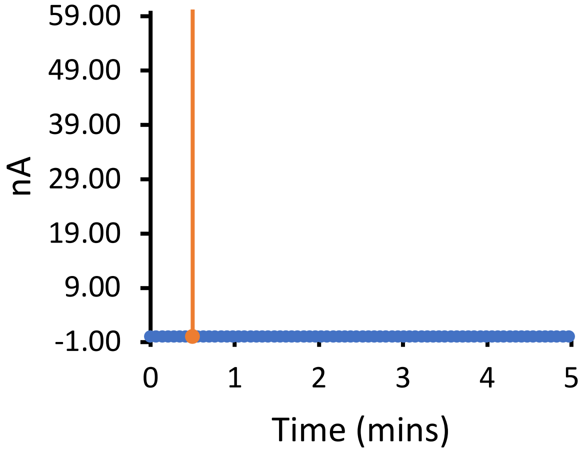

*Figure S31 - Experimental recording 2 of a DPhPC bilayer at 100 mV, addition of **1** (0.10 mM) after 30 seconds indicated by the orange line.*

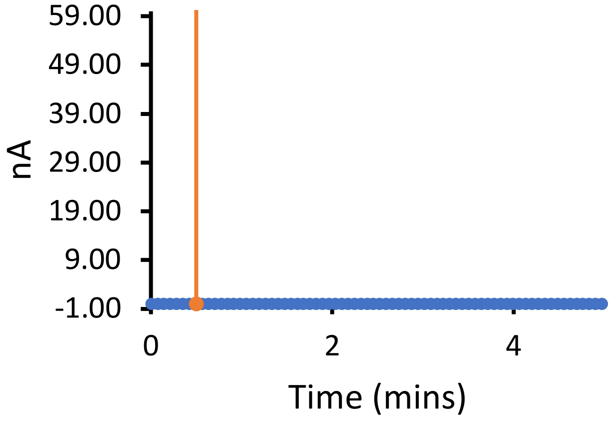

*Figure S32 - Experimental recording 3 of a DPhPC bilayer at 100 mV, addition of **1** (0.10 mM) after 30 seconds indicated by the orange line.*

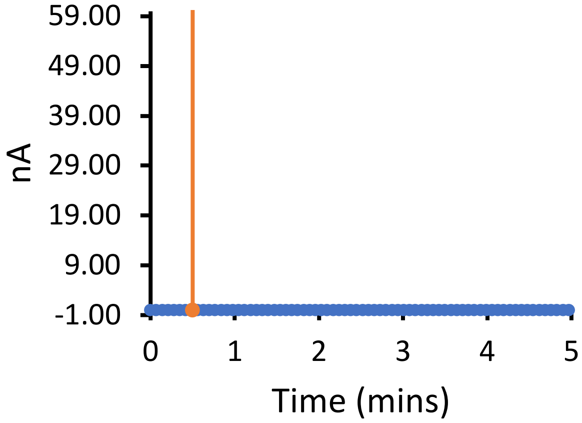

*Figure S33 – Experimental recording 1 of a DPhPC bilayer at 100 mV, addition of **4** (0.01 mM) at 0 seconds and **1** (0.10 mM) after 30 seconds indicated by the orange line.*

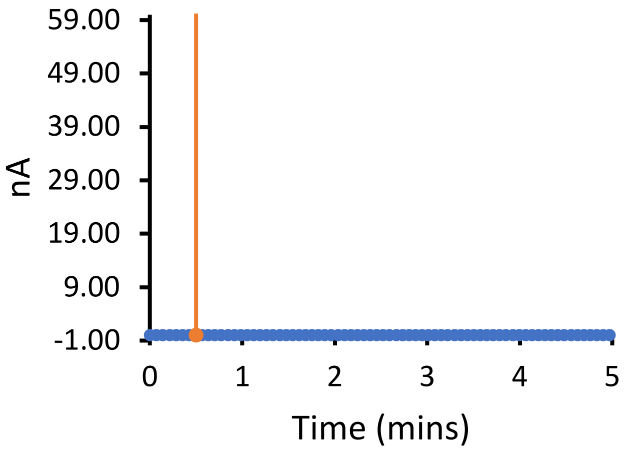

*Figure S34 - Experimental recording 2 of a DPhPC bilayer at 100 mV, addition of **4** (0.01 mM) at 0 seconds and **1** (0.10 mM) after 30 seconds indicated by the orange line.*

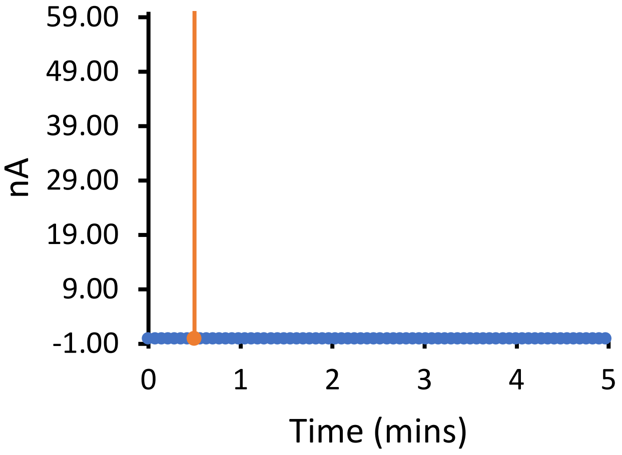

*Figure S35 - Experimental recording 3 of a DPhPC bilayer at 100 mV, addition of **4** (0.01 mM) at 0 seconds and **1** (0.10 mM) after 30 seconds indicated by the orange line.*

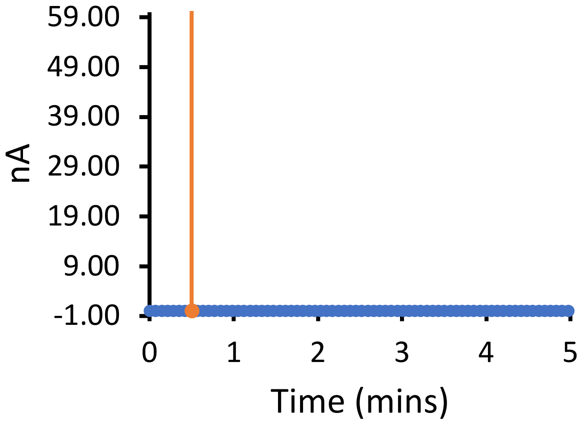

*Figure S36 – Experimental recording 1 of a DPhPC bilayer at 100 mV, addition of **1** (0.25 mM) after 30 seconds indicated by the orange line.*

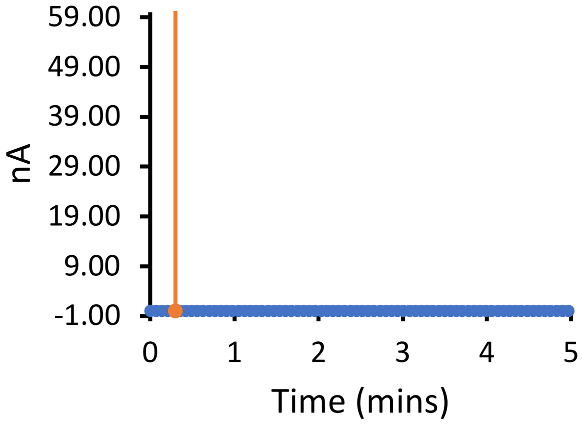

*Figure S37 - Experimental recording 2 of a DPhPC bilayer at 100 mV, addition of **1** (0.25 mM) after 30 seconds indicated by the orange line.*

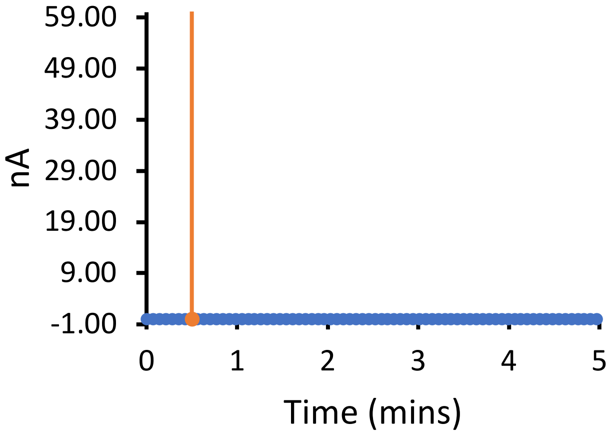

*Figure S38 - Experimental recording 3 of a DPhPC bilayer at 100 mV, addition of **1** (0.25 mM) after 30 seconds indicated by the orange line.*

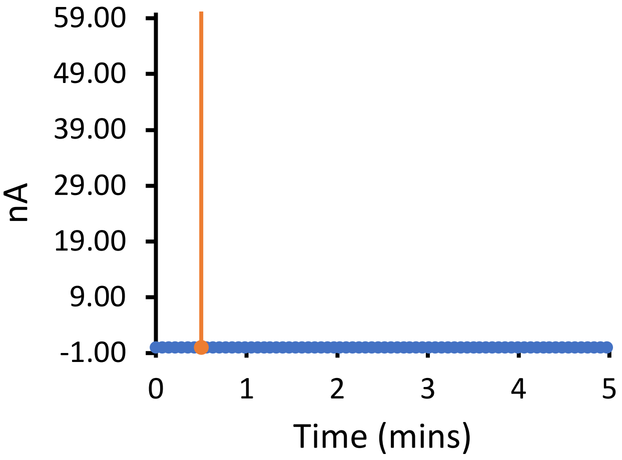

*Figure S39 – Experimental recording 1 of a DPhPC bilayer at 100 mV, addition of **4** (0.01 mM) at 0 seconds and **1** (0.25 mM) after 30 seconds indicated by the orange line.*

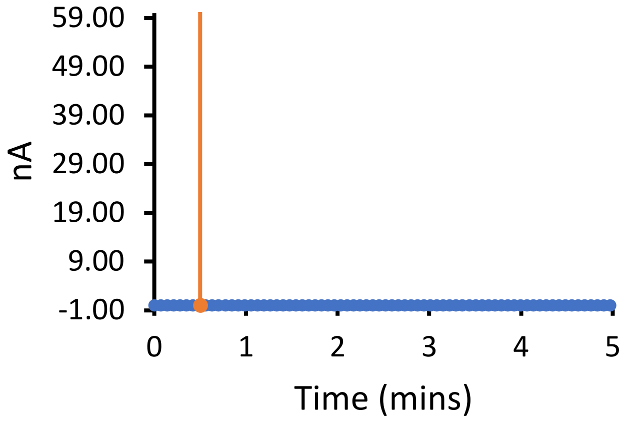

*Figure S40 - Experimental recording 2 of a DPhPC bilayer at 100 mV, addition of **4** (0.01 mM) at 0 seconds and **1** (0.25 mM) after 30 seconds indicated by the orange line.*

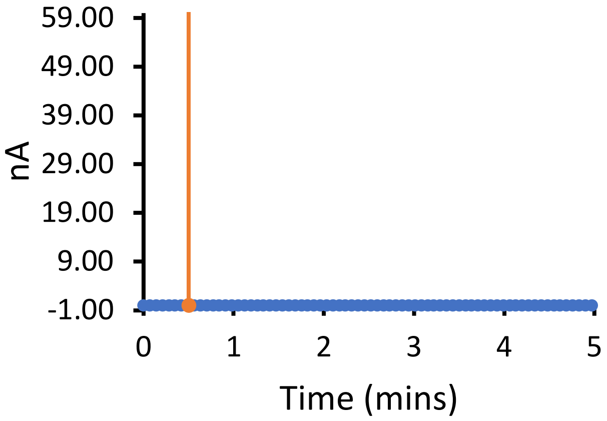

*Figure S41 - Experimental recording 3 of a DPhPC bilayer at 100 mV, addition of **4** (0.01 mM) at 0 seconds and **1** (0.25 mM) after 30 seconds indicated by the orange line.*

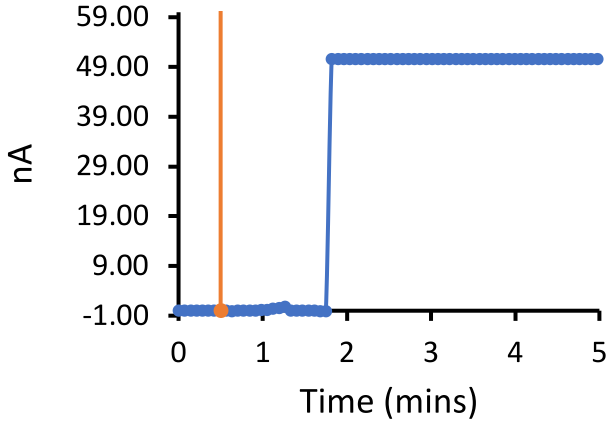

*Figure S42 – Experimental recording 1 of a DPhPC bilayer at 100 mV, addition of **1** (0.375 mM) after 30 seconds indicated by the orange line.*

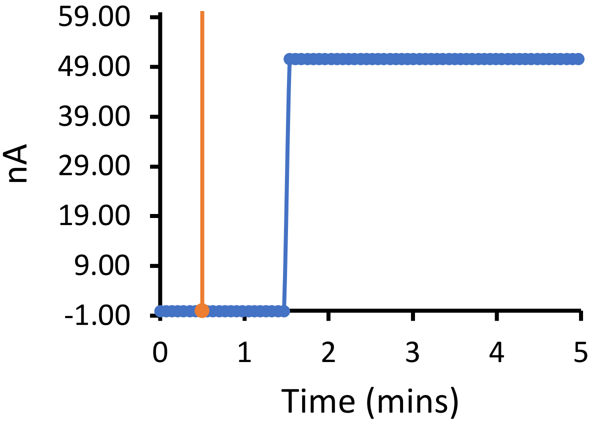

*Figure S43 - Experimental recording 2 of a DPhPC bilayer at 100 mV, addition of **1** (0.375 mM) after 30 seconds indicated by the orange line.*

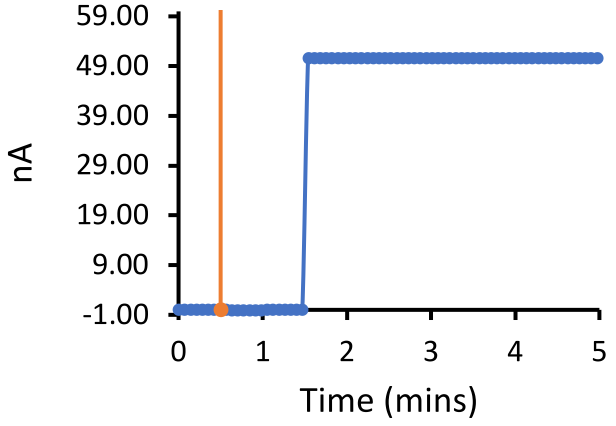

*Figure S44 - Experimental recording 3 of a DPhPC bilayer at 100 mV, addition of **1** (0.375 mM) after 30 seconds indicated by the orange line.*

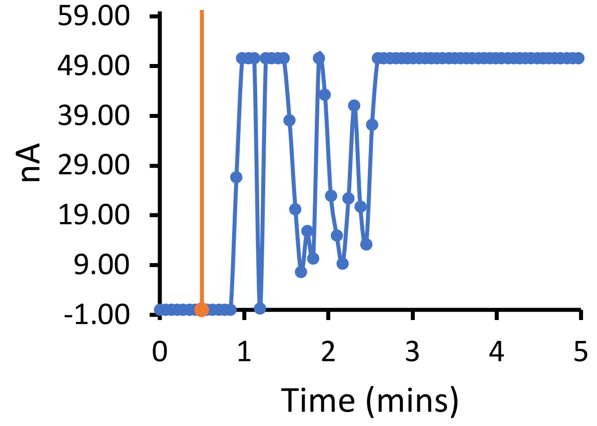

*Figure S45 – Experimental recording 1 of a DPhPC bilayer at 100 mV, addition of **4** (0.01 mM) at 0 seconds and **1** (0.375 mM) after 30 seconds indicated by the orange line.*

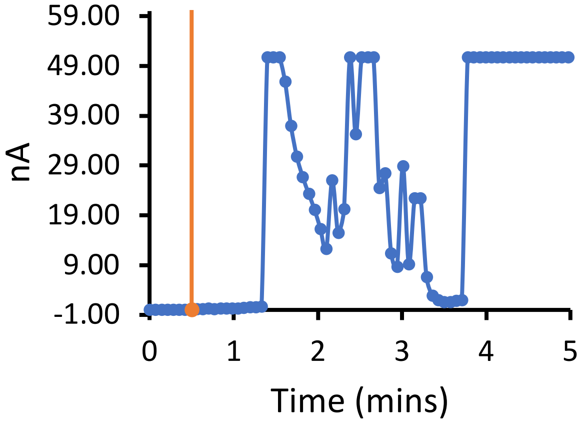

*Figure S46 - Experimental recording 2 of a DPhPC bilayer at 100 mV, addition of **4** (0.01 mM) at 0 seconds and **1** (0.375 mM) after 30 seconds indicated by the orange line.*

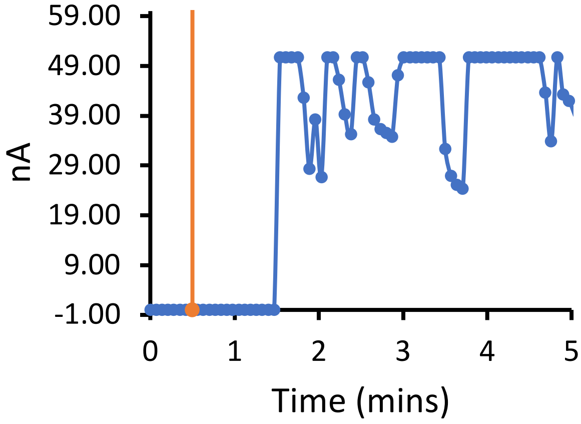

*Figure S47 - Experimental recording 3 of a DPhPC bilayer at 100 mV, addition of **4** (0.01 mM) at 0 seconds and **1** (0.375 mM) after 30 seconds indicated by the orange line.*

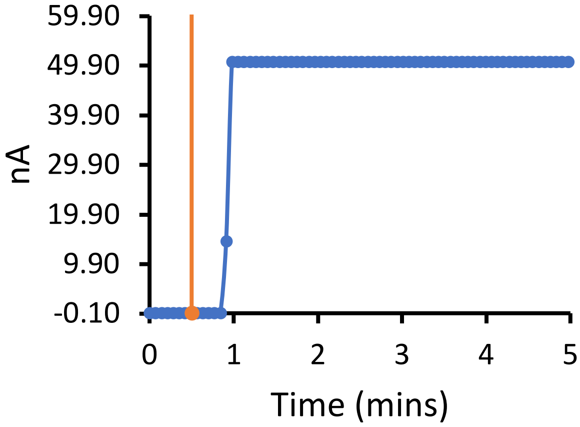

*Figure S48 – Experimental recording 1 of a DPhPC bilayer at 100 mV, addition of **1** (0.5 mM) after 30 seconds indicated by the orange line.*

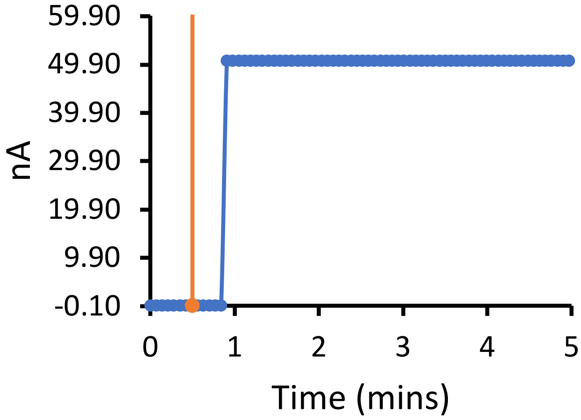

*Figure S49 - Experimental recording 2 of a DPhPC bilayer at 100 mV, addition of **1** (0.5 mM) after 30 seconds indicated by the orange line.*

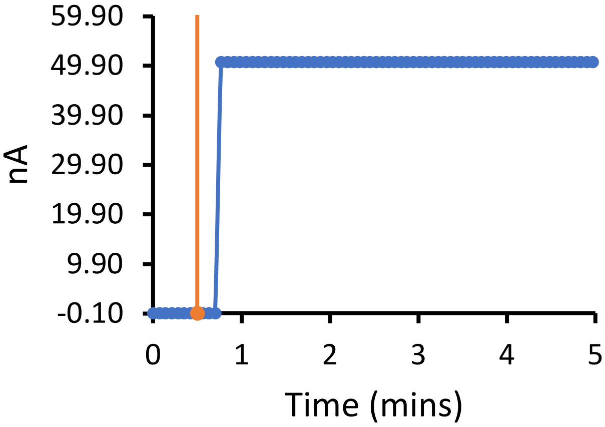

*Figure S50 - Experimental recording 3 of a DPhPC bilayer at 100 mV, addition of **1** (0.5 mM) after 30 seconds indicated by the orange line.*

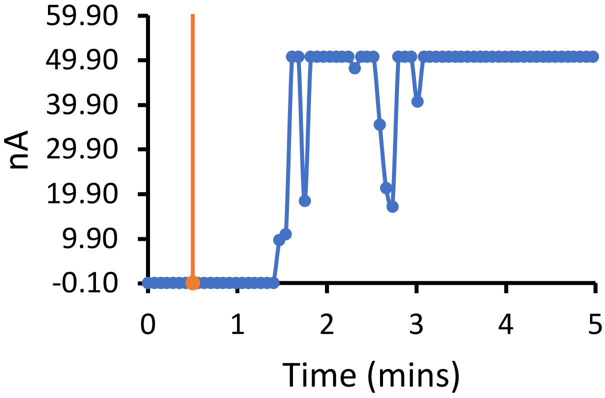

*Figure S51 – Experimental recording 1 of a DPhPC bilayer at 100 mV, addition of **4** (0.01 mM) at 0 seconds and **1** (0.50 mM) after 30 seconds indicated by the orange line.*

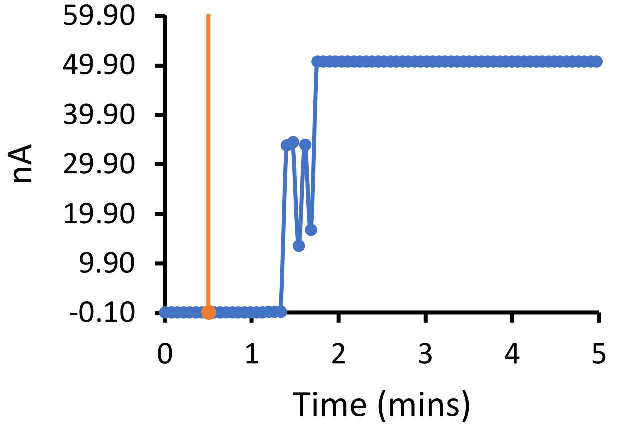

*Figure S52 - Experimental recording 2 of a DPhPC bilayer at 100 mV, addition of **4** (0.01 mM) at 0 seconds and **1** (0.50 mM) after 30 seconds indicated by the orange line.*

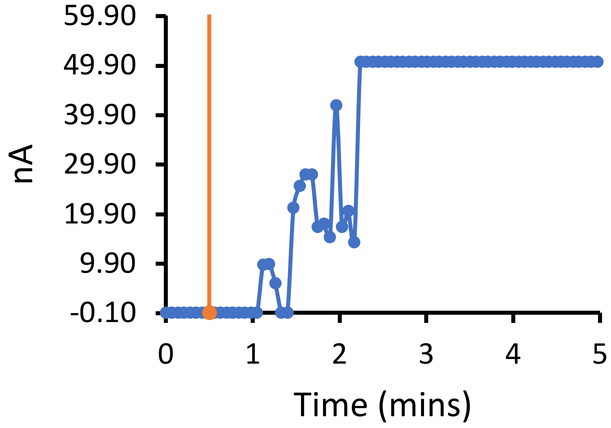

*Figure S53 - Experimental recording 3 of a DPhPC bilayer at 100 mV, addition of **4** (0.01 mM) at 0 seconds and **1** (0.50 mM) after 30 seconds indicated by the orange line.*

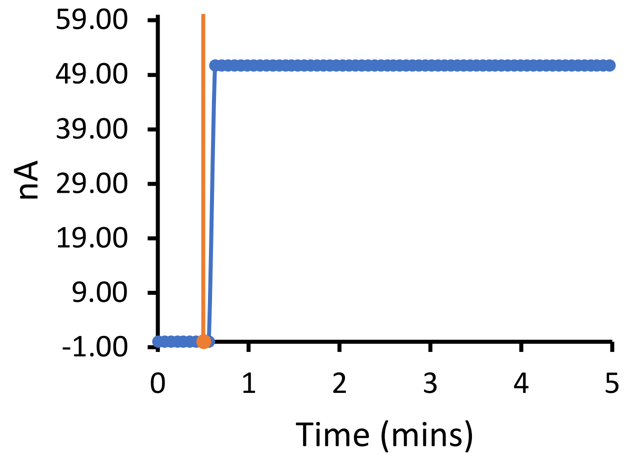

*Figure S54 – Experimental recording 1 of a DPhPC bilayer at 100 mV, addition of **1** (1.00 mM) after 30 seconds indicated by the orange line.*

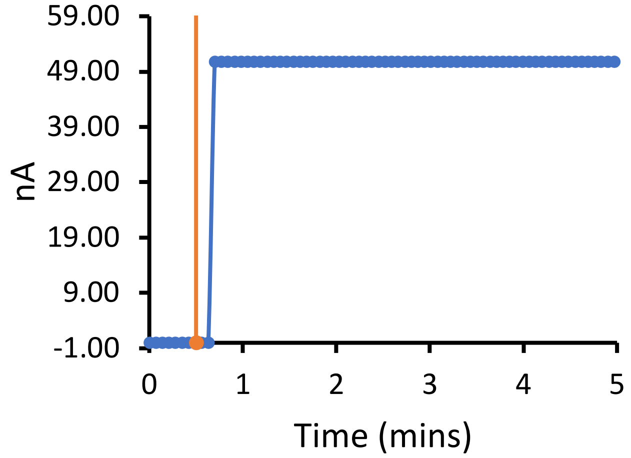

*Figure S55 - Experimental recording 2 of a DPhPC bilayer at 100 mV, addition of **1** (1.00 mM) after 30 seconds indicated by The orange line.*

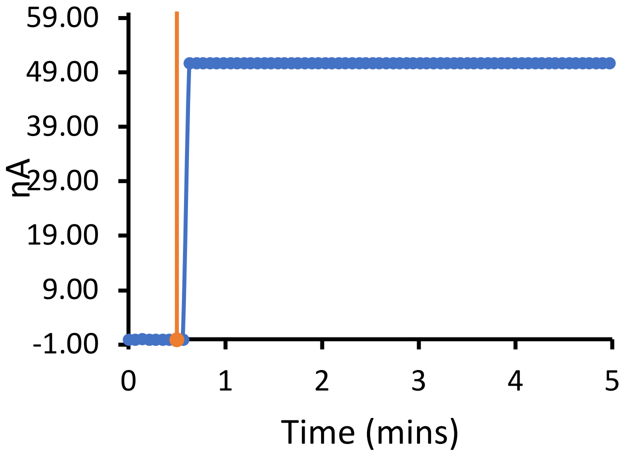

*Figure S56 - Experimental recording 3 of a DPhPC bilayer at 100 mV, addition of **1** (1.00 mM) after 30 seconds indicated by the orange line.*

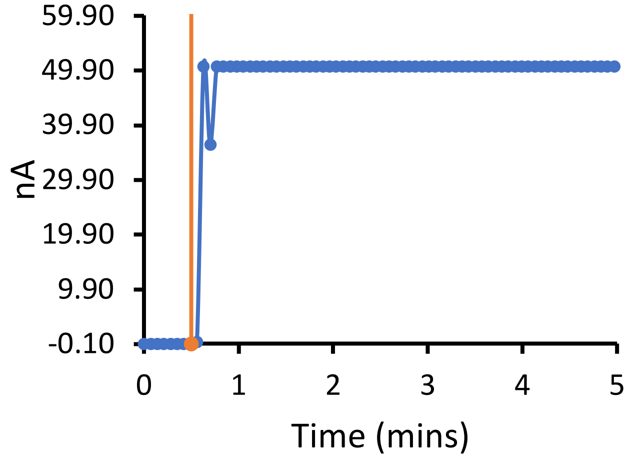

*Figure S57 – Experimental recording 1 of a DPhPC bilayer at 100 mV, addition of **4** (0.01 mM) at 0 seconds and **1** (1.00 mM) after 30 seconds indicated by the orange line.*

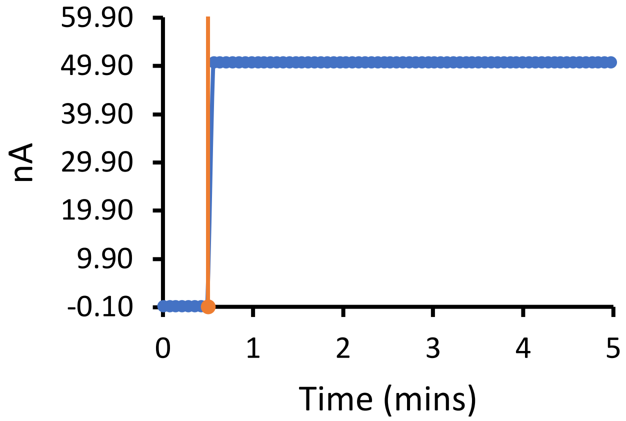

*Figure S58 - Experimental recording 2 of a DPhPC bilayer at 100 mV, addition of **4** (0.01 mM) at 0 seconds and **1** (1.00 mM) after 30 seconds indicated by the orange line.*

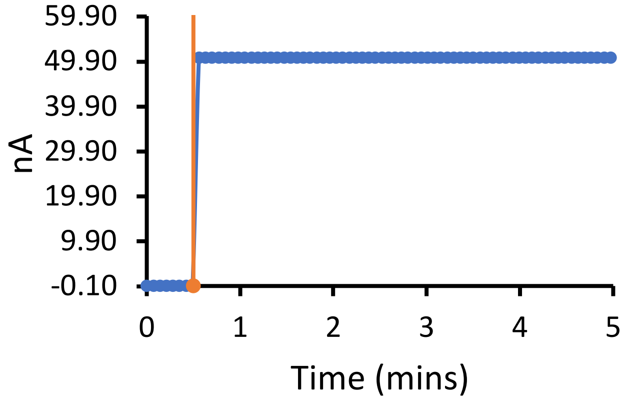

*Figure S59 – Experimental recording 3 of a DPhPC bilayer at 100 mV, addition of **4** (0.01 mM) at 0 seconds and **1** (1.00 mM) after 30 seconds indicated by the orange line.*

<u>SSA **2**</u>

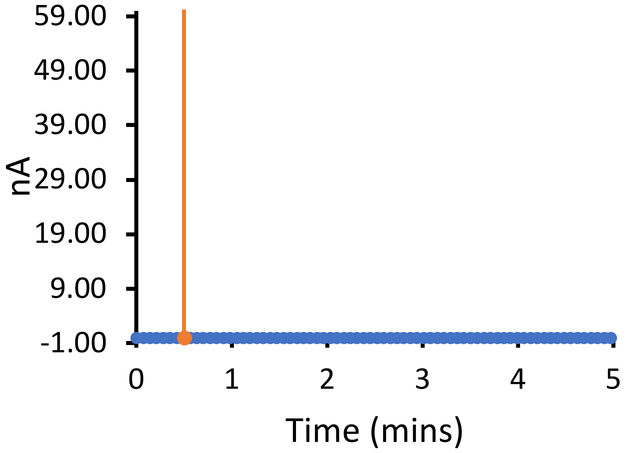

*Figure S60 – Experimental recording 1 of a DPhPC bilayer at 100 mV, addition of **2** (0.1 mM) after 30 seconds indicated by the orange line.*

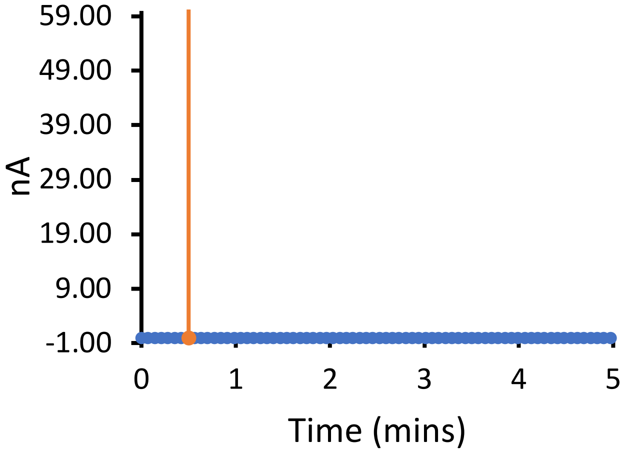

*Figure S61 - Experimental recording 2 of a DPhPC bilayer at 100 mV, addition of **2** (0.1 mM) after 30 seconds indicated by the orange line.*

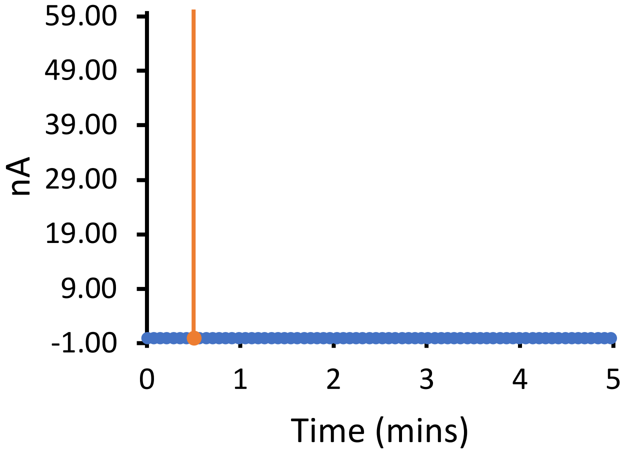

*Figure S62 - Experimental recording 3 of a DPhPC bilayer at 100 mV, addition of **2** (0.10 mM) after 30 seconds indicated by the orange line.*

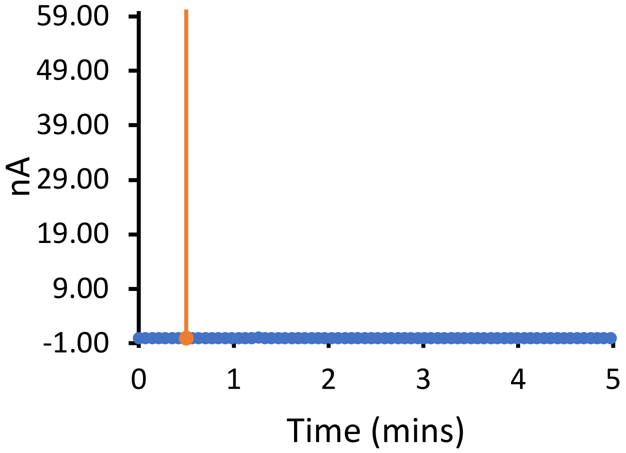

*Figure S63 – Experimental recording 1 of a DPhPC bilayer at 100 mV, addition of **4** (0.01 mM) at 0 seconds and **2** (0.10 mM) after 30 seconds indicated by the orange line.*

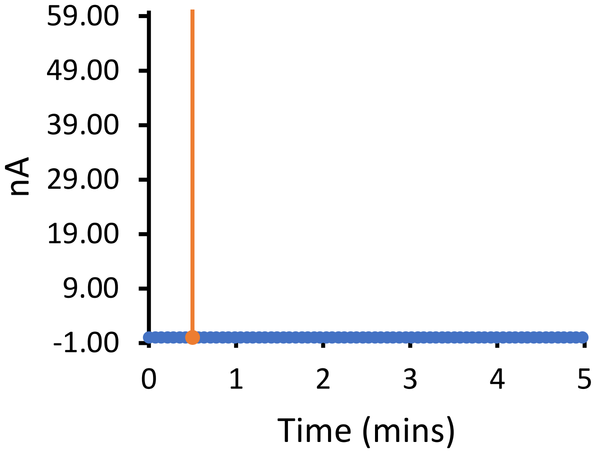

*Figure S64 - Experimental recording 2 of a DPhPC bilayer at 100 mV, addition of **4** (0.01 mM) at 0 seconds and **2** (0.10 mM) after 30 seconds indicated by the orange line.*

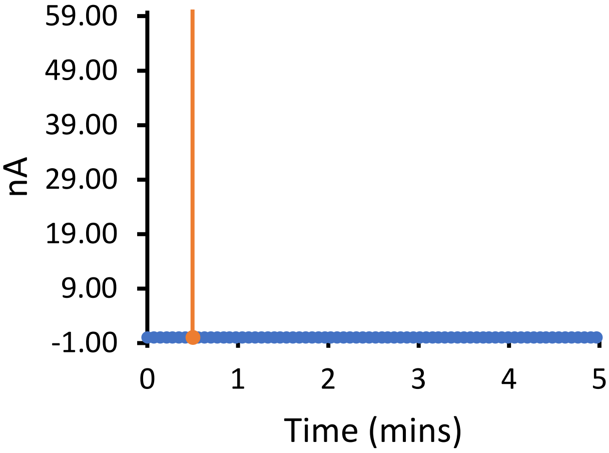

*Figure S65 – Experimental recording 3 of a DPhPC bilayer at 100 mV, addition of **4** (0.01 mM) at 0 seconds and **2** (0.10 mM) after 30 seconds indicated by the orange line.*

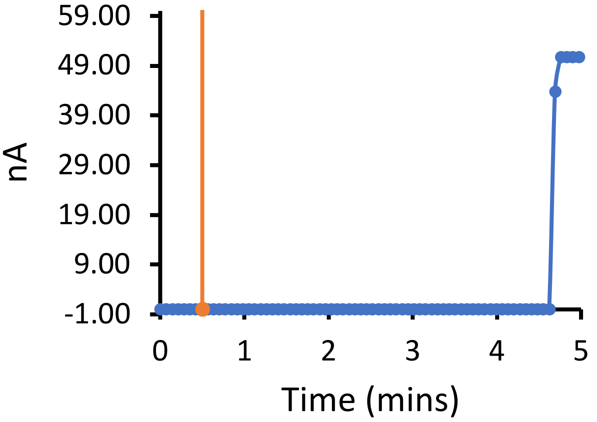

*Figure S66 – Experimental recording 1 of a DPhPC bilayer at 100 mV, addition of **2** (0.25 mM) after 30 seconds indicated by the orange line.*

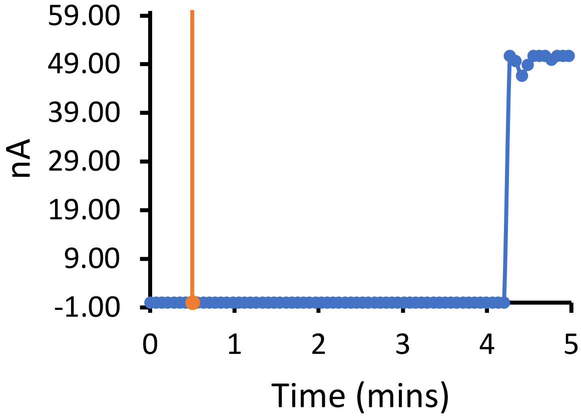

*Figure S67 - Experimental recording 2 of a DPhPC bilayer at 100 mV, addition of **2** (0.25 mM) after 30 seconds indicated by the orange line.*

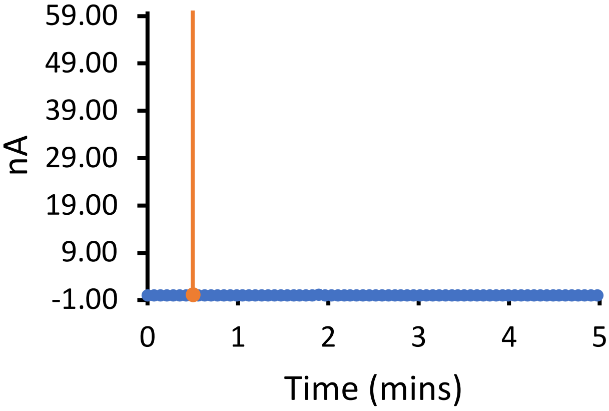

*Figure S68 - Experimental recording 3 of a DPhPC bilayer at 100 mV, addition of **2** (0.25 mM) after 30 seconds indicated by the orange line.*

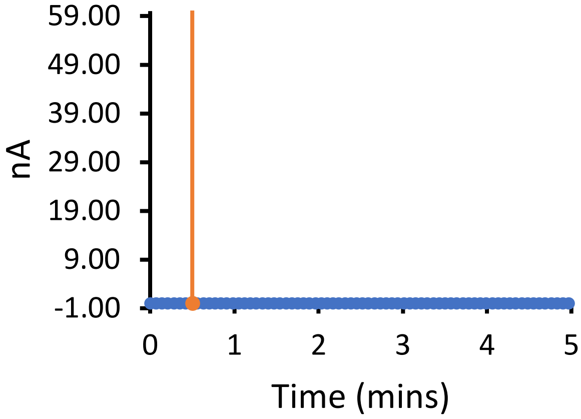

*Figure S69 – Experimental recording 1 of a DPhPC bilayer at 100 mV, addition of **4** (0.01 mM) at 0 seconds and **2** (0.25 mM) after 30 seconds indicated by the orange line.*

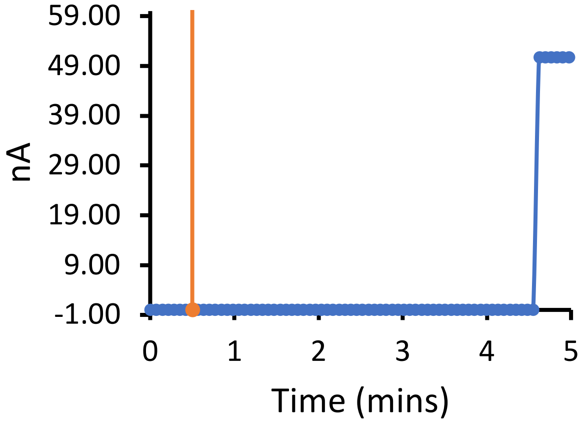

*Figure S70 - Experimental recording 2 of a DPhPC bilayer at 100 mV, addition of **4** (0.01 mM) at 0 seconds and **2** (0.25 mM) after 30 seconds indicated by the orange line.*

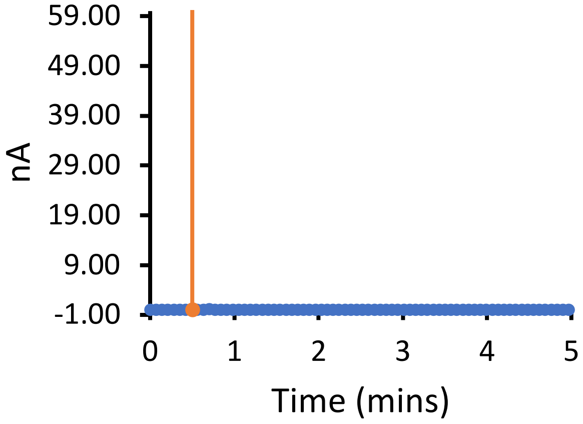

*Figure S71 - Experimental recording 3 of a DPhPC bilayer at 100 mV, addition of **4** (0.01 mM) at 0 seconds and **2** (0.25 mM) after 30 seconds indicated by the orange line.*

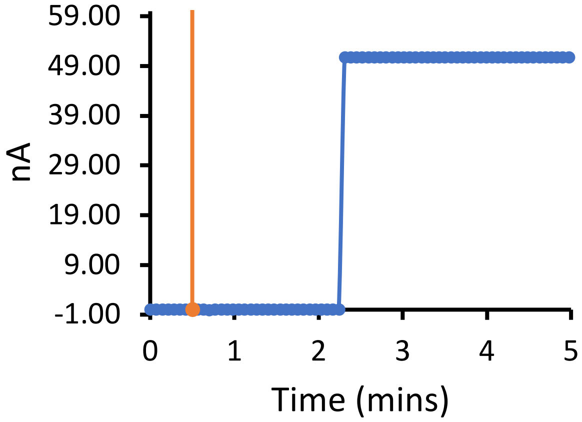

*Figure S72 – Experimental recording 1 of a DPhPC bilayer at 100 mV, addition of **2** (0.375 mM) after 30 seconds indicated by the orange line.*

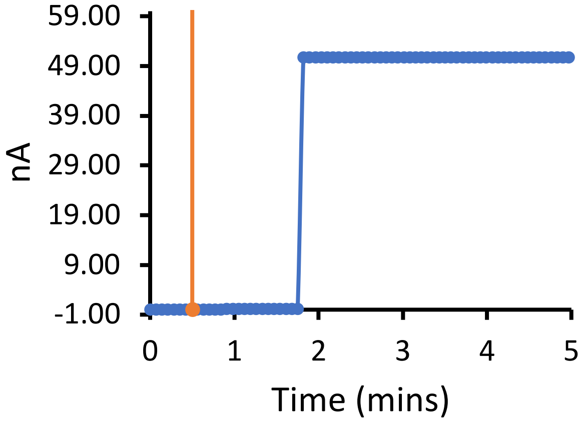

*Figure S73 — Experimental recording 2 of a DPhPC bilayer at 100 mV, addition of **2** (0.375 mM) after 30 seconds indicated by the orange line.*

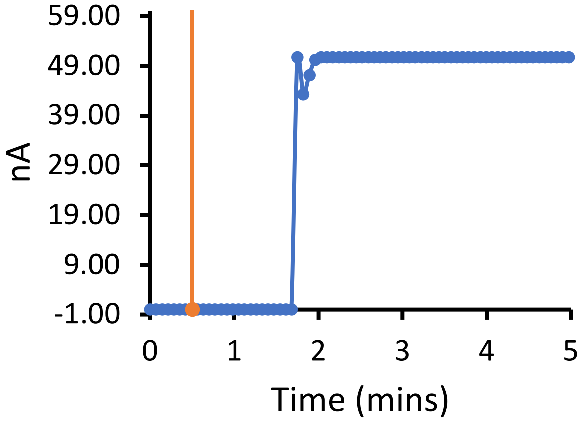

*Figure S74 – Experimental recording 3 of a DPhPC bilayer at 100 mV, addition of **2** (0.375 mM) after 30 seconds indicated by the orange line.*

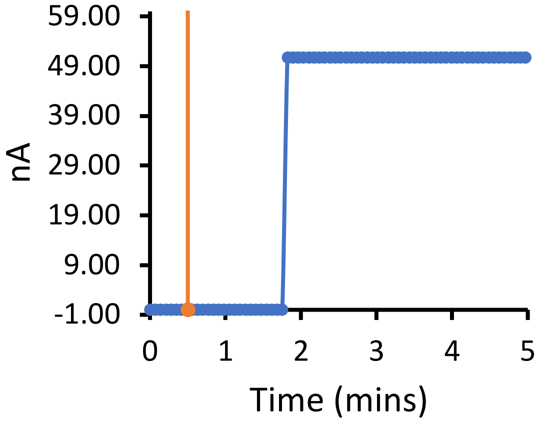

*Figure S75 – Experimental recording 1 of a DPhPC bilayer at 100 mV, addition of **4** (0.01 mM) at 0 seconds and **2** (0.375 mM) after 30 seconds indicated by the orange line.*

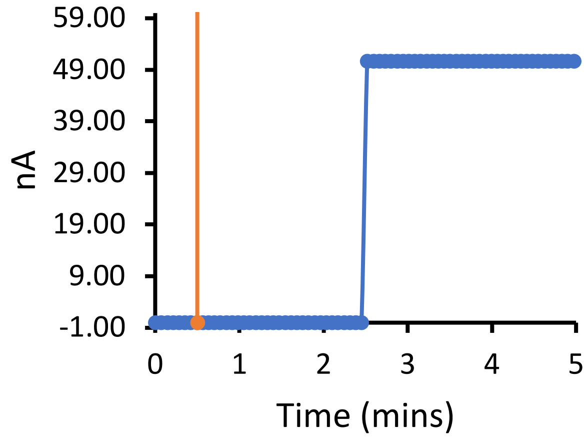

*Figure S76 - Experimental recording 2 of a DPhPC bilayer at 100 mV, addition of **4** (0.01 mM) at 0 seconds and **2** (0.375 mM) after 30 seconds indicated by the orange line.*

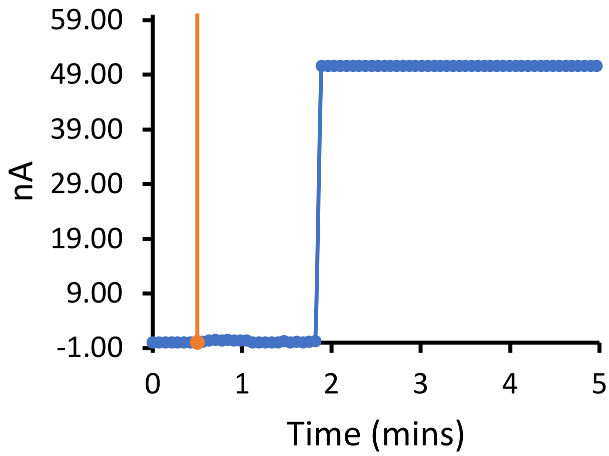

*Figure S77 - Experimental recording 3 of a DPhPC bilayer at 100 mV, addition of **4** (0.01 mM) at 0 seconds and **2** (0.375 mM) after 30 seconds indicated by the orange line.*

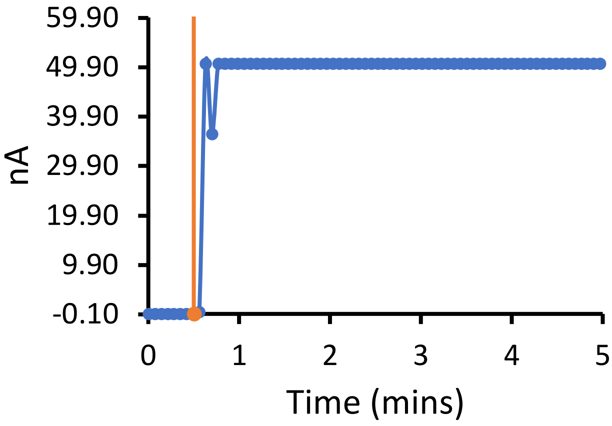

*Figure S78 – Experimental recording 1 of a DPhPC bilayer at 100 mV, addition of **2** (1.00 mM) after 30 seconds indicated by the orange line.*

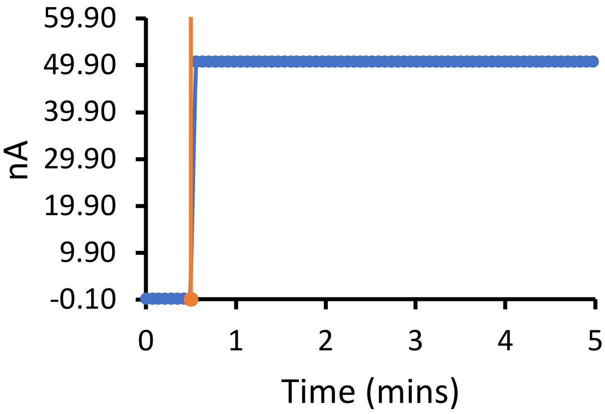

*Figure S79 - Experimental recording 2 of a DPhPC bilayer at 100 mV, addition of **2** (1.00 mM) after 30 seconds indicated by the orange line.*

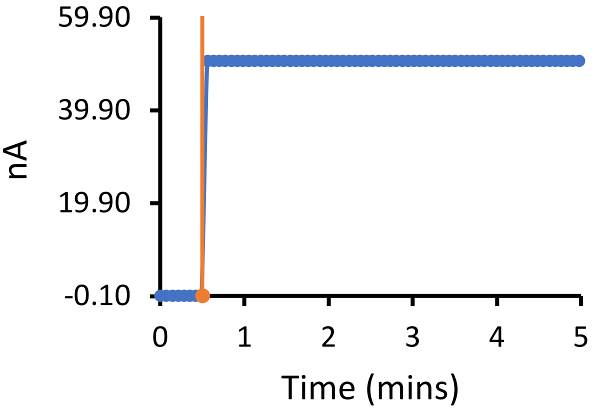

*Figure S80 - Experimental recording 3 of a DPhPC bilayer at 100 mV, addition of **2** (1.00 mM) after 30 seconds indicated by the orange line.*

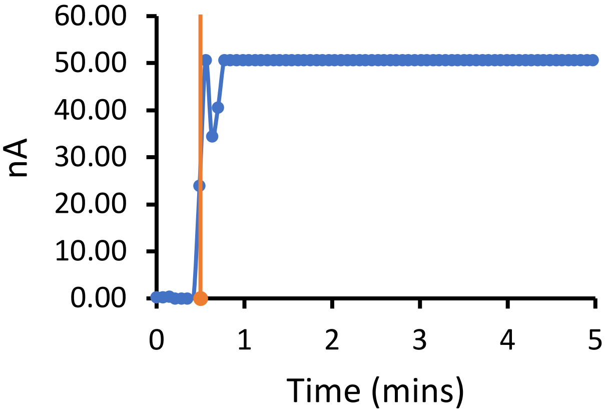

*Figure S81 – Experimental recording 1 of a DPhPC bilayer at 100 mV,addition of **4** (0.01 mM) at 0 seconds and **2** (1.00 mM) after 30 seconds indicated by the orange line.*

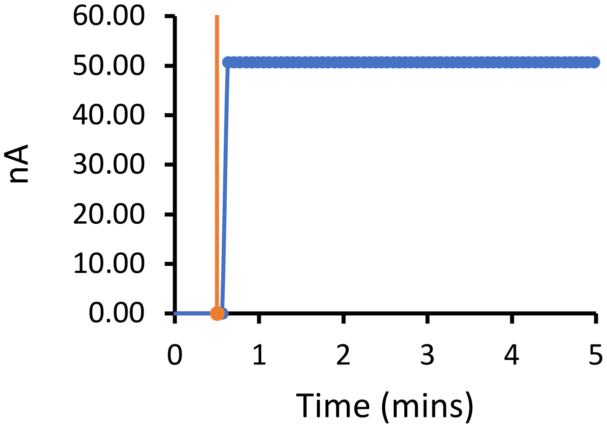

*Figure S82 - Experimental recording 2 of a DPhPC bilayer at 100 mV, addition of **4** (0.01 mM) at 0 seconds and **2** (1.00 mM) after 30 seconds indicated by the orange line.*

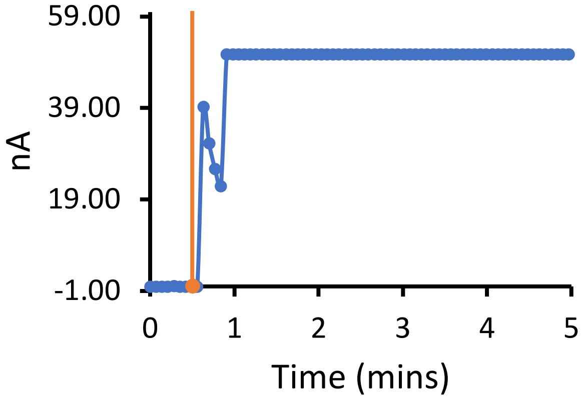

*Figure S83 - Experimental recording 3 of a DPhPC bilayer at 100 mV, addition of **4** (0.01 mM) at 0 seconds and **2** (1.00 mM) after 30 seconds indicated by the orange line.*

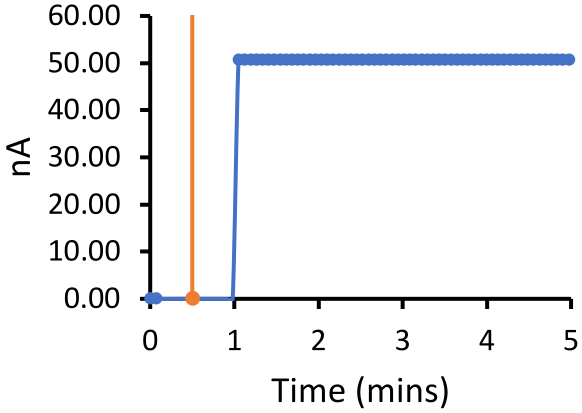

Figure S84 – Experimental recording 1 of a DPhPC bilayer at 100 mV, addition of 2 (0.5 mM) after 30 seconds indicated by the orange line.

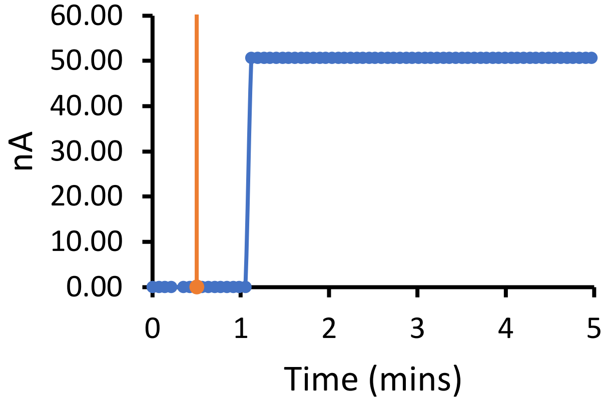

Figure S85 - Experimental recording 2 of a DPhPC bilayer at 100 mV, addition of 2 (0.5 mM) after 30 seconds indicated by the orange line.

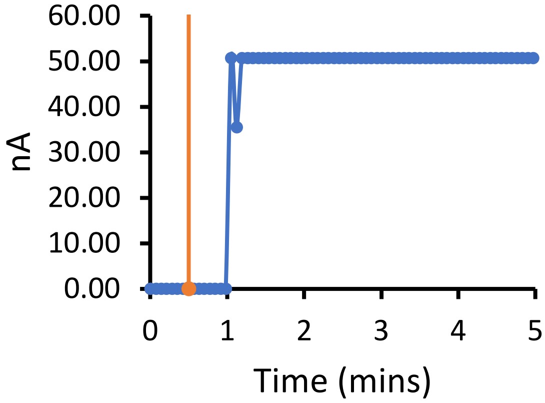

Figure S86 - Experimental recording 3 of a DPhPC bilayer at 100 mV, addition of 2 (0.5 mM) after 30 seconds indicated by the orange line.

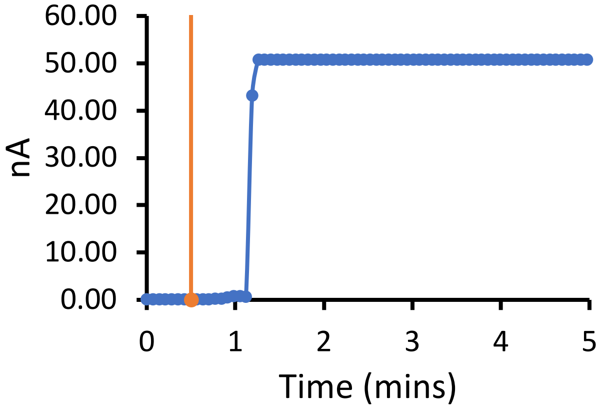

Figure S87 – Experimental recording 1 of a DPhPC bilayer at 100 mV, addition of 4 (0.01 mM) at 0 seconds and 2 (0.5 mM) after 30 seconds indicated by the orange line

SSA **3**

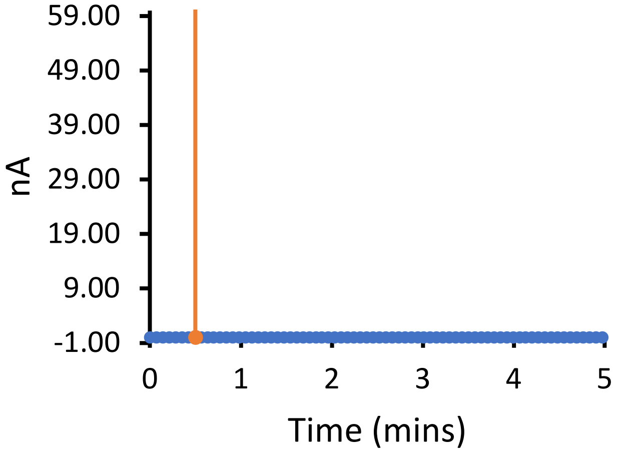

Figure S88 – Experimental recording 1 of a DPhPC bilayer at 100 mV, addition of ***3*** *(0.1 mM) after 30 seconds indicated by the orange line.*

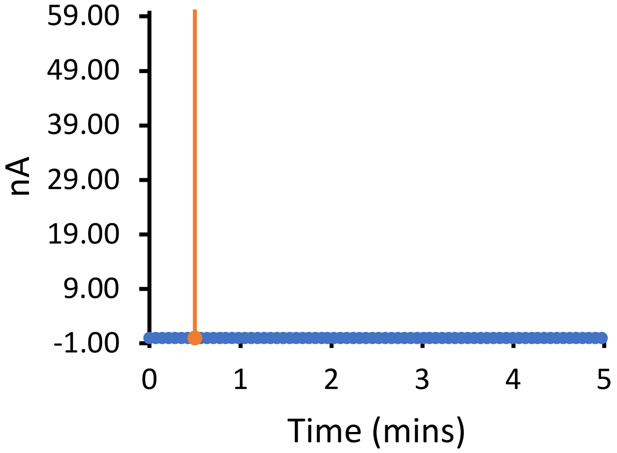

Figure S89 - Experimental recording 2 of a DPhPC bilayer at 100 mV, addition of ***3*** *(0.1 mM) after 30 seconds indicated by the orange line.*

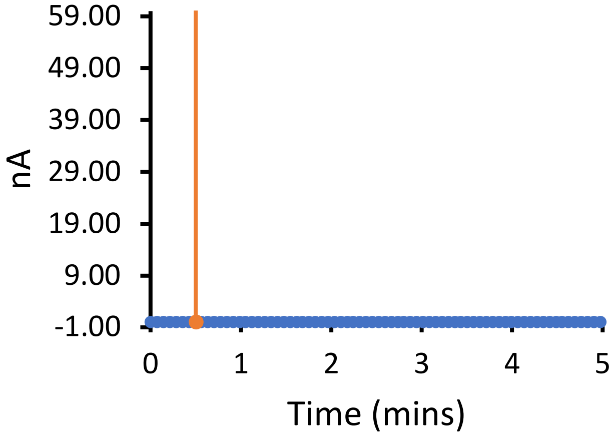

Figure 90 - Experimental recording 3 of a DPhPC bilayer at 100 mV, addition of ***3*** *(0.1 mM) after 30 seconds indicated by the orange line.*

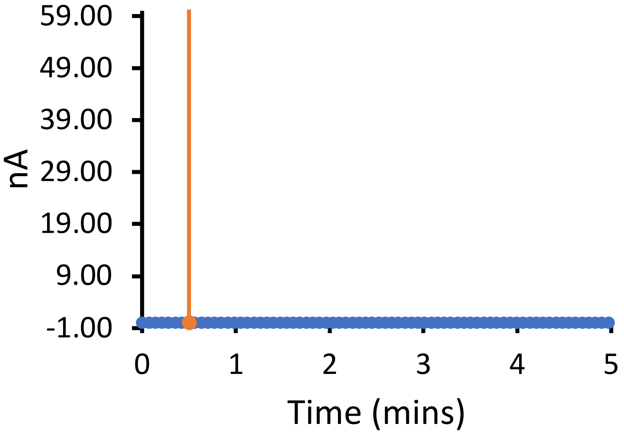

Figure S91 – Experimental recording 1 of a DPhPC bilayer at 100 mV, addition of ***4*** *(0.01 mM) at 0 seconds and* ***3*** *(0.10 mM) after 30 seconds indicated by the orange line.*

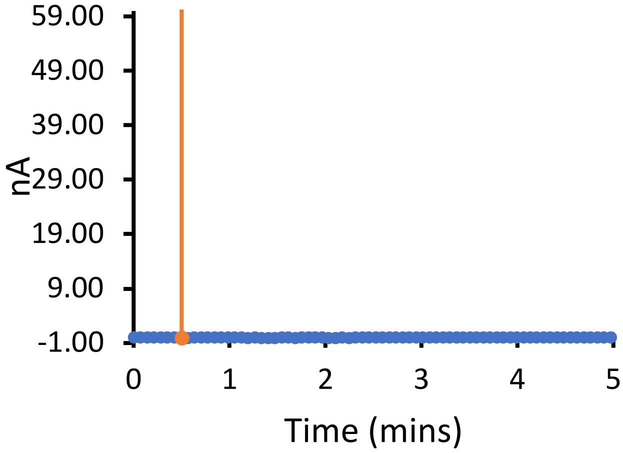

Figure S92- Experimental recording 2 of a DPhPC bilayer at 100 mV, addition of ***4*** *(0.01 mM) at 0 seconds and* ***3*** *(0.10 mM) after 30 seconds indicated by the orange line.*

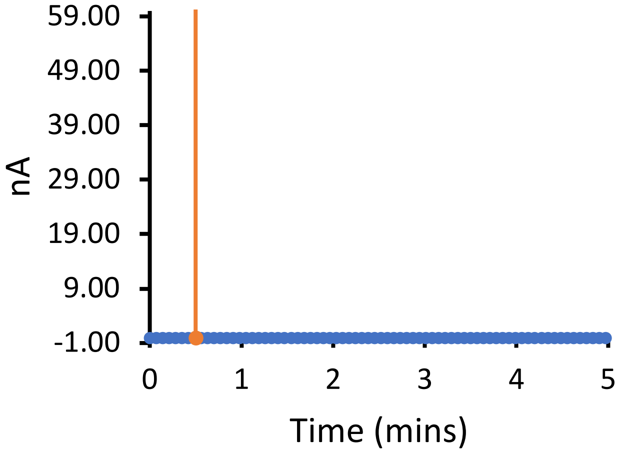

Figure S93 – Experimental recording 3 of a DPhPC bilayer at 100 mV, addition of ***4*** *(0.01 mM) at 0 seconds and* ***3*** *(0.10 mM) after 30 seconds indicated by the orange line.*

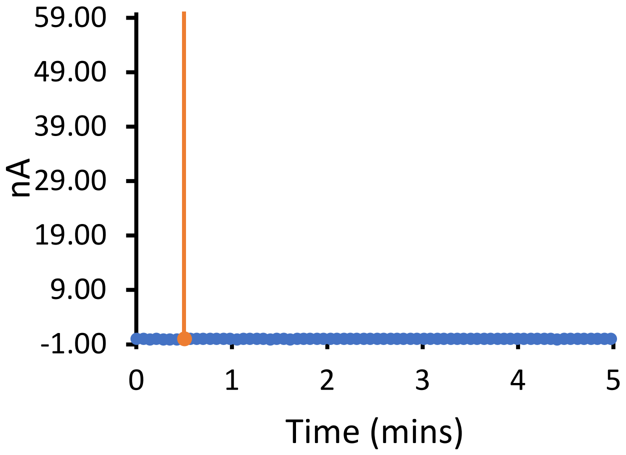

*Figure S94 – Experimental recording 1 of a DPhPC bilayer at 100 mV, addition of **3** (0.25 mM) after 30 seconds indicated by the orange line.*

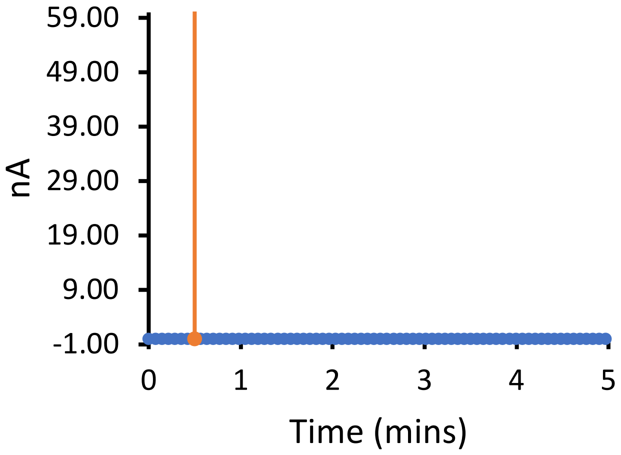

*Figure S95 - Experimental recording 2 of a DPhPC bilayer at 100 mV, addition of **3** (0.25 mM) after 30 seconds indicated by the orange line.*

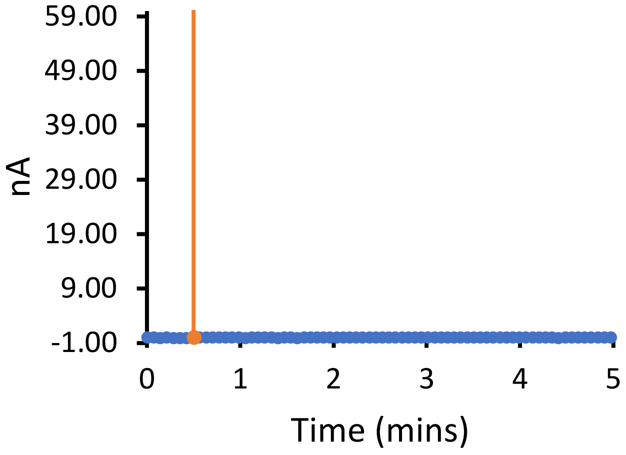

*Figure S96 - Experimental recording 3 of a DPhPC bilayer at 100 mV, addition of **3** (0.25 mM) after 30 seconds indicated by the orange line.*

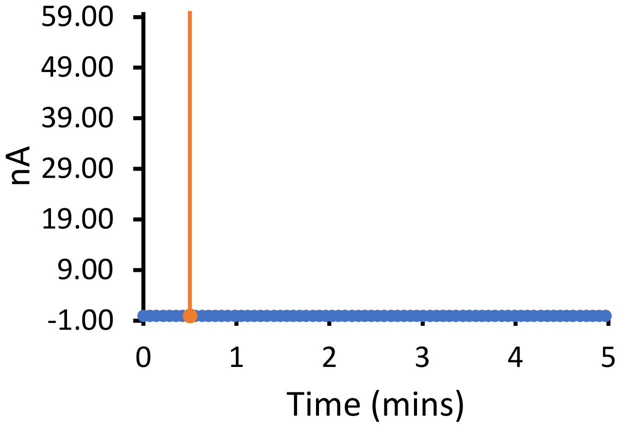

*Figure S97 – Experimental recording 1 of a DPhPC bilayer at 100 mV, addition of **4** (0.01 mM) at 0 seconds and **3** (0.25 mM) after 30 seconds indicated by the orange line.*

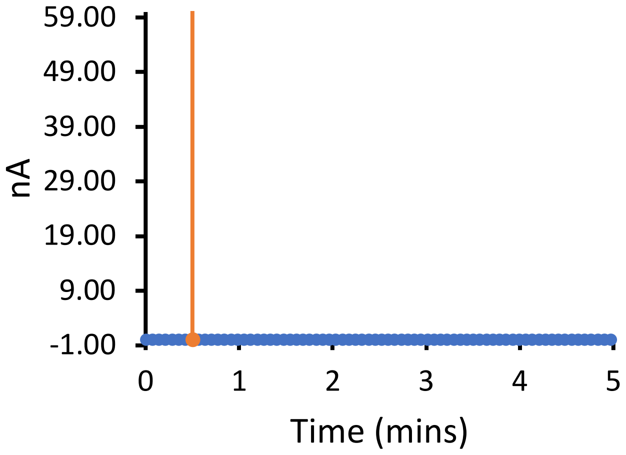

*Figure S98 - Experimental recording 2 of a DPhPC bilayer at 100 mV, addition of **4** (0.01 mM) at 0 seconds and **3** (0.25 mM) after 30 seconds indicated by the orange line.*

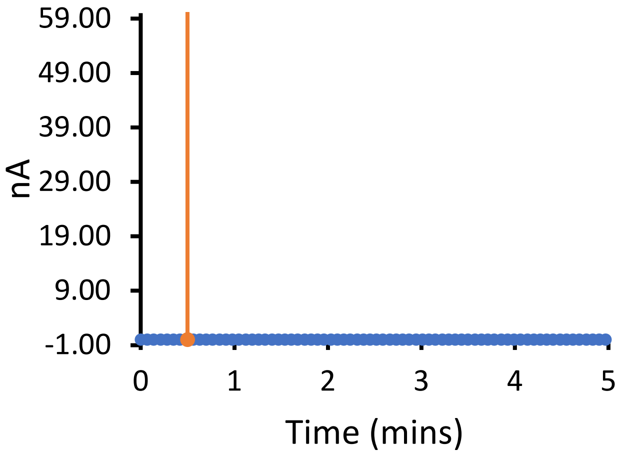

*Figure S99 - Experimental recording 3 of a DPhPC bilayer at 100 mV, addition of **4** (0.01 mM) at 0 seconds and **3** (0.25 mM) after 30 seconds indicated by the orange line.*

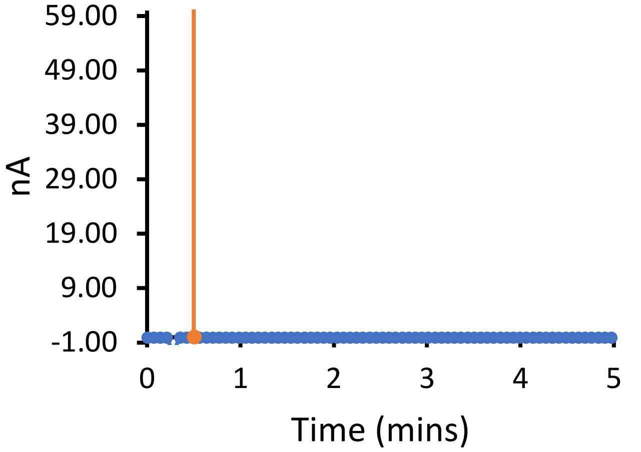

*Figure S100 – Experimental recording 1 of a DPhPC bilayer at 100 mV, addition of **3** (0.375 mM) after 30 seconds indicated by the orange line.*

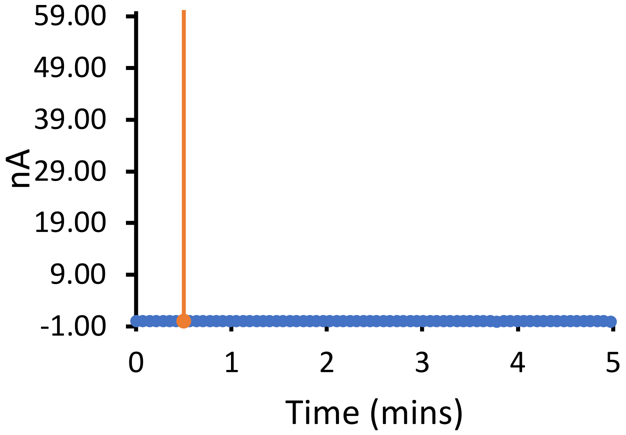

*Figure S101- Experimental recording 2 of a DPhPC bilayer at 100 mV, addition of **3** (0.375 mM) after 30 seconds indicated by the orange line.*

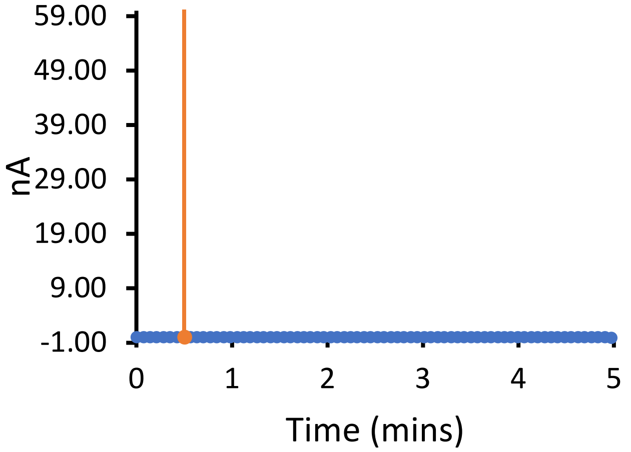

*Figure S102 - Experimental recording 3 of a DPhPC bilayer at 100 mV, addition of **3** (0.375 mM) after 30 seconds indicated by the orange line.*

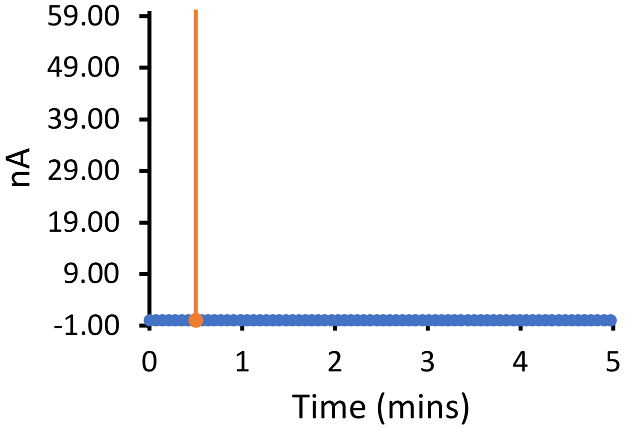

*Figure S103 – Experimental recording 1 of a DPhPC bilayer at 100 mV, addition of **4** (0.01 mM) at 0 seconds and **3** (0.375 mM) after 30 seconds indicated by the orange line.*

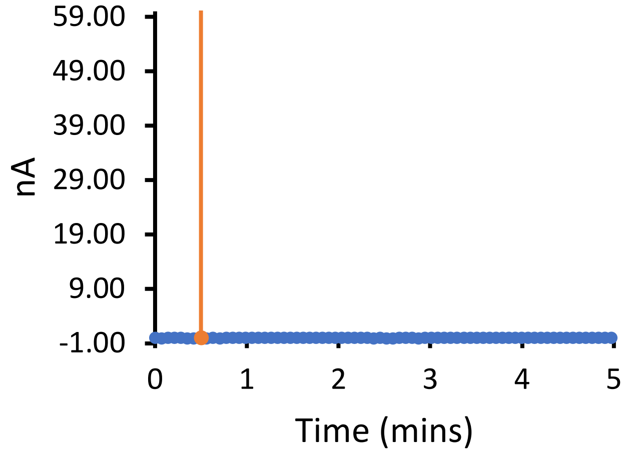

*Figure S104 - Experimental recording 2 of a DPhPC bilayer at 100 mV, addition of **4** (0.01 mM) at 0 seconds and **3** (0.375 mM) after 30 seconds indicated by the orange line.*

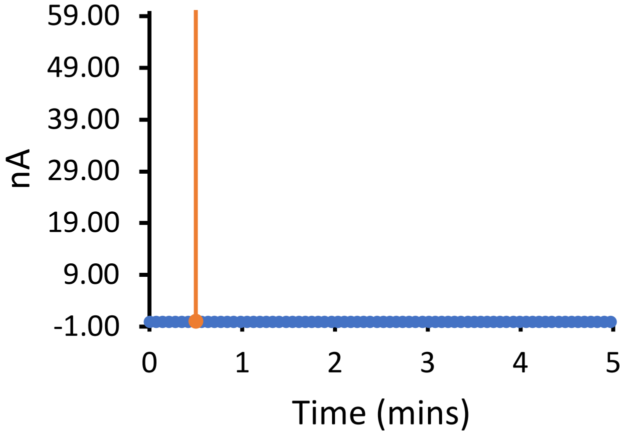

*Figure S105 - Experimental recording 3 of a DPhPC bilayer at 100 mV, addition of **4** (0.01 mM) at 0 seconds and **3** (0.375 mM) after 30 seconds indicated by the orange line.*

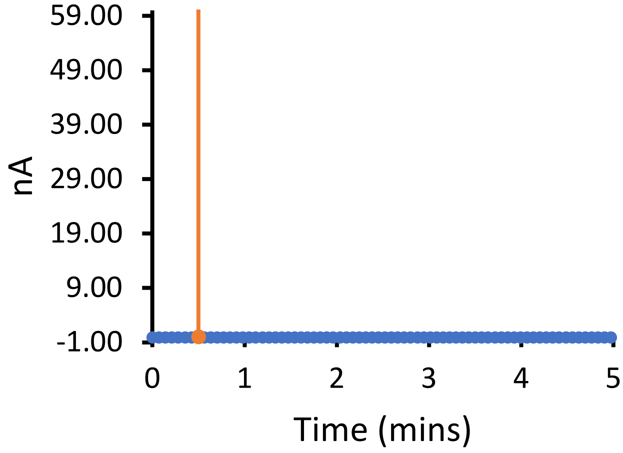

*Figure S106 – Experimental recording 1 of a DPhPC bilayer at 100 mV, addition of **3** (0.50 mM) after 30 seconds indicated by the orange line.*

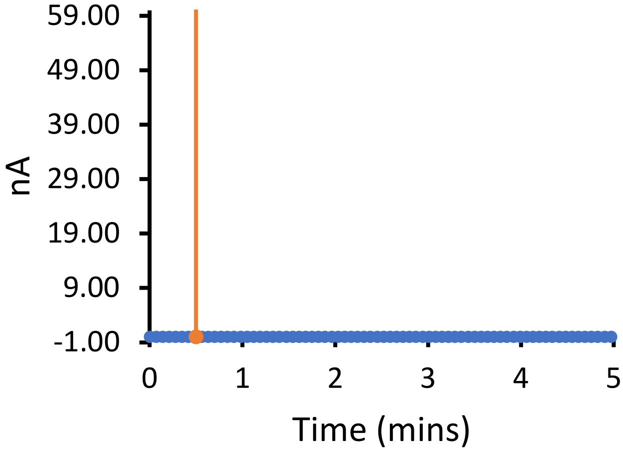

*Figure S107 - Experimental recording 2 of a DPhPC bilayer at 100 mV, addition of **3** (0.50 mM) after 30 seconds indicated by the orange line.*

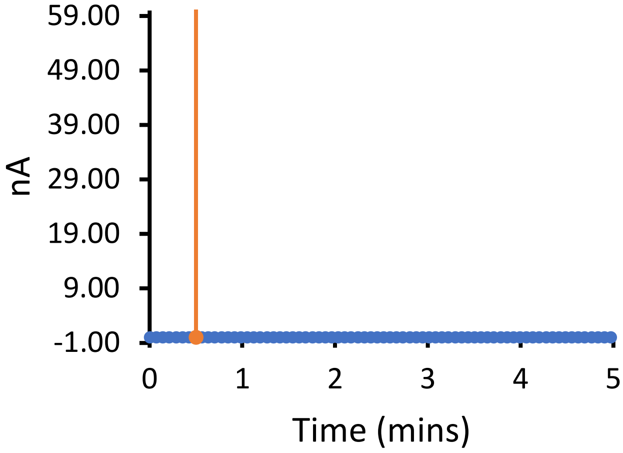

*Figure S108 - Experimental recording 3 of a DPhPC bilayer at 100 mV, addition of **3** (0.50 mM) after 30 seconds indicated by the orange line.*

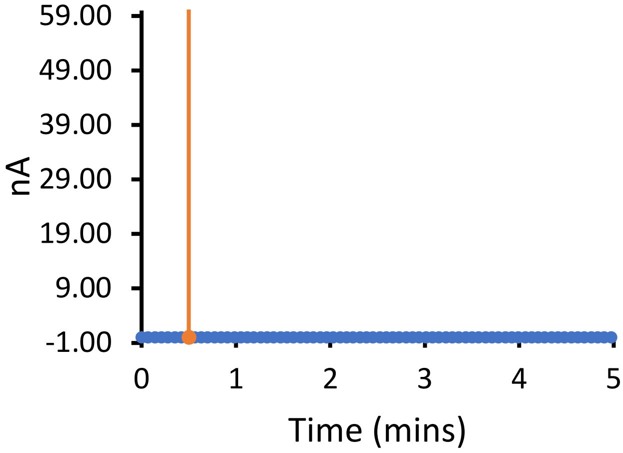

*Figure S109 – Experimental recording 1 of a DPhPC bilayer at 100 mV, addition of **4** (0.01 mM) at 0 seconds and **3** (0.50 mM) after 30 seconds indicated by the orange line.*

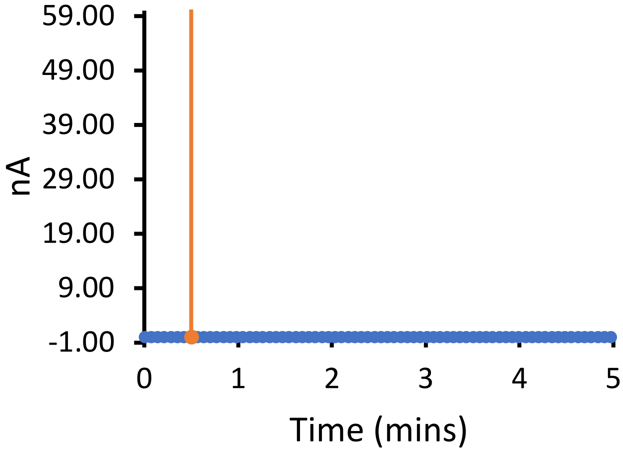

*Figure S110 - Experimental recording 2 of a DPhPC bilayer at 100 mV, addition of **4** (0.01 mM) at 0 seconds and **3** (0.50 mM) after 30 seconds indicated by the orange line.*

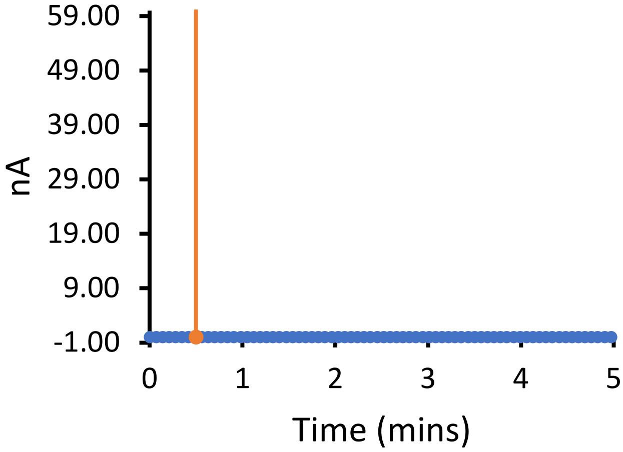

*Figure S111 – Experimental recording 3 of a DPhPC bilayer at 100 mV, addition of **4** (0.01 mM) at 0 seconds and **3** (0.50 mM) after 30 seconds indicated by the orange line.*

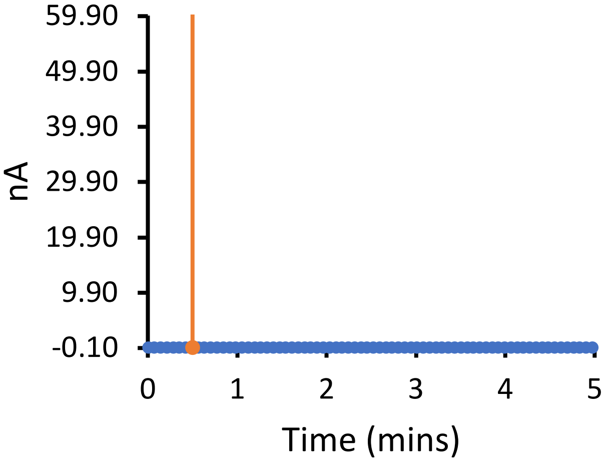

Figure S112 – Experimental recording 1 of a DPhPC bilayer at 100 mV, addition of ***3*** *(1.00 mM) after 30 seconds indicated by the orange line.*

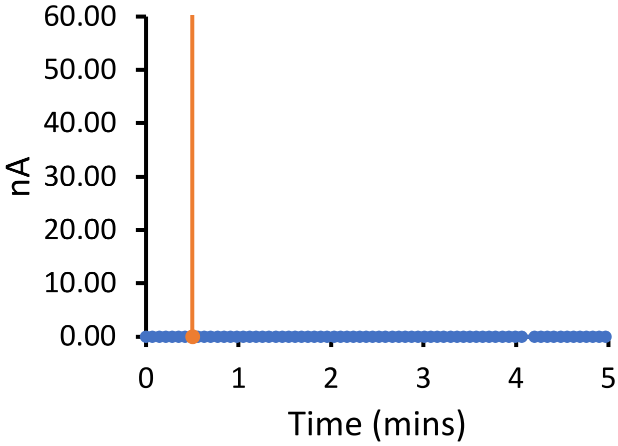

Figure S113 - Experimental recording 2 of a DPhPC bilayer at 100 mV, addition of ***3*** *(1.00 mM) after 30 seconds indicated by th orange line.*

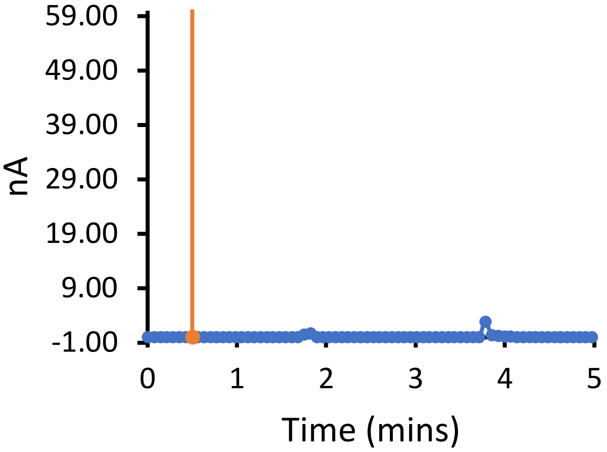

Figure S114 - Experimental recording 3 of a DPhPC bilayer at 100 mV, addition of ***3*** *(1.00 mM) after 30 seconds indicated by the orange line.*

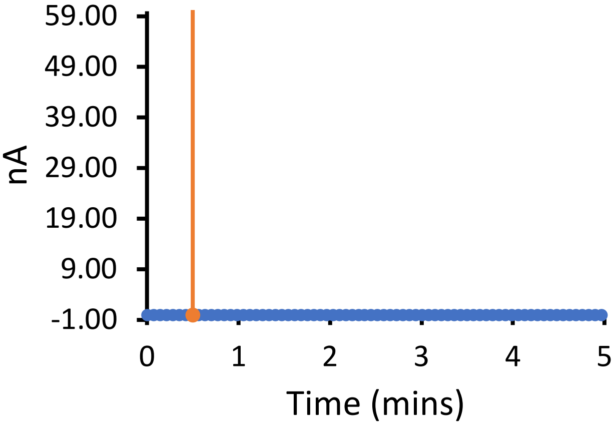

Figure S115 – Experimental recording 1 of a DPhPC bilayer at 100 mV, addition of ***4*** *(0.01 mM) at 0 seconds and* ***3*** *(1.00 mM) after 30 seconds indicated by the orange line.*

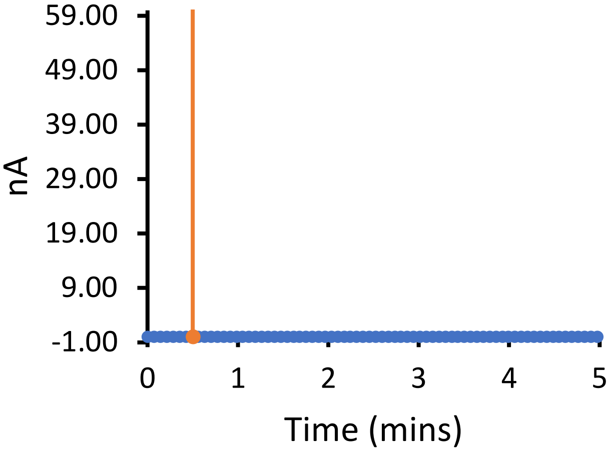

Figure S116 - Experimental recording 2 of a DPhPC bilayer at 100 mV, addition of ***4*** *(0.01 mM) at 0 seconds and* ***3*** *(1.00 mM) after 30 seconds indicated by the orange line.*

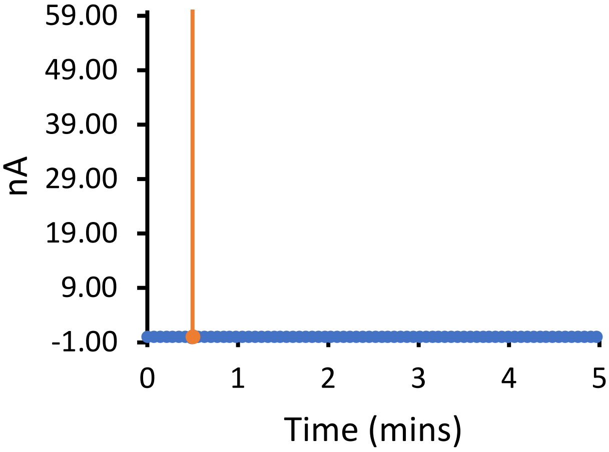

Figure S117 - Experimental recording 3 of a DPhPC bilayer at 100 mV, addition of ***4*** *(0.01 mM) at 0 seconds and* ***3*** *(1.00 mM) after 30 seconds indicated by the orange line.*

S4.3 Patch clamp data discussion

We found that adding SSAs **1**–**3** and **4** alone from stocks solutions of lower concentrations (≤ 0.25 mM) alone did not produce an elevated current recording over the experimental timeframe (see figures S26–28, S29–40, S59–67 and S87–107). At higher concentrations of SSA, we observed bilayer rupture (figures S41–-43 and S71–73), identified by a constant current above the limit of detection of the instrument (50 nA) and the live information on bilayer rupture provided by the instrument. Here, the SSA acts as a simple surfactant, effectively destroying the phospholipid planar bilayer. However, under specific conditions, the addition of SSA **1** and anionophore **4** together produced an erratic, elevated current flow across the clamped portion of the bilayer, until eventual bilayer rupture was observed ≈ 210 seconds after SSA addition (figures S44–46 and main text figure 3). The elevated current prior to bilayer rupture was identified by the fluctuation of current above and below the 50 nA limit of detection; any eventual full bilayer rupture was identified by a loss of fluctuation and a constant current > 50 nA, in addition to the live information on bilayer rupture provided by the instrument.

We therefore suggest that SSA **1** operates in combination with anionophore **4** to initiate concentration dependent bulk ion transport events.

S5 References

1 L. J. White, N. J. Wells, L. R. Blackholly, H. J. Shepherd, B. Wilson, G. P. Bustone, T. J. Runacres and J. R. Hiscock, *Chem. Sci.*, 2017, **8**, 7620–7630.

2 S. J. Moore, M. Wenzel, M. E. Light, R. Morley, S. J. Bradberry, P. Gómez-Iglesias, V. Soto-Cerrato, R. Pérez-Tomás and P. A. Gale, *Chem. Sci*., 2012, **3**, 2501–2509.

3 Y.-H. Li, S. Zheng, Y.-M. Legrand, A. Giles, a. Van der Lee and M. Barboiu, *Angew. Chem. Int. Ed*. 2018, **57**, 10520–10524.

4 S.-H. Park, I. Hwang, D. A. McNaughton, A. J. Kinross, E. N. W. Howe, Q. He, S. Xiong, M. D. Kilde, V. M. Lynch, P. A. Gale, J. L. Sessler and I. Shin, *Chem*, 2021, **7**, 3325–3339.

5 J. E. Boles, C. Bennett, J. Baker, K. L. F. Hilton, H. A. Kotak, E. R. Clark, Y. Long, L. J. White, H. Y. Lai, C. K. Hind, J. M. Sutton, M. D. Garrett, A. Cheasty, J. L. Ortega-Roldan, M. Charles, C. J. E. Haynes and J. R. Hiscock, *under consideration*, 2022.
